# Global exact optimisations for chloroplast structural haplotype scaffolding

**DOI:** 10.1186/s13015-023-00243-1

**Published:** 2024-02-06

**Authors:** Victor Epain, Rumen Andonov

**Affiliations:** GenScale, Centre Inria de l’Université de Rennes, IRISA, 263 Avenue Général Leclerc, 35700 Rennes, France

**Keywords:** Genome assembly, Inverted repeats, Integer linear programming, NP-complete problem

## Abstract

**Background:**

Scaffolding is an intermediate stage of fragment assembly. It consists in orienting and ordering the contigs obtained by the assembly of the sequencing reads. In the general case, the problem has been largely studied with the use of distances data between the contigs. Here we focus on a dedicated scaffolding for the chloroplast genomes. As these genomes are small, circular and with few specific repeats, numerous approaches have been proposed to assemble them. However, their specificities have not been sufficiently exploited.

**Results:**

We give a new formulation for the scaffolding in the case of chloroplast genomes as a discrete optimisation problem, that we prove the decision version to be $\mathcal{NP}$-Complete. We take advantage of the knowledge of chloroplast genomes and succeed in expressing the relationships between a few specific genomic repeats in mathematical constraints. Our approach is independent of the distances and adopts a genomic regions view, with the priority on scaffolding the repeats first. In this way, we encode the structural haplotype issue in order to retrieve several genome forms that coexist in the same chloroplast cell. To solve exactly the optimisation problem, we develop an integer linear program that we implement in Python3 package khloraascaf. We test it on synthetic data to investigate its performance behaviour and its robustness against several chosen difficulties.

**Conclusions:**

We succeed to model biological knowledge on genomic structures to scaffold chloroplast genomes. Our results suggest that modelling genomic regions is sufficient for scaffolding repeats and is suitable for finding several solutions corresponding to several genome forms.

## Background

DNA molecule is a support of living mechanism information found in all the living organisms. Its sequence can be seen as a word on the alphabet $\Sigma _{nuc}{} = \left\{ A, C, G, T\right\}$. Genetic, genomic and epigenetic analysis lead to combinatorial problems that need to be solved by computing approaches and thus require to pass from the DNA molecule to a word representation in a computer. This process is described as sequencing the molecule using a sequencing technology.

### The scaffolding in the process of fragment assembly

Current technologies are not yet able to read an entire DNA molecule. They output a huge amount of small overlapping erroneous DNA sequences, named *reads*. Moreover, due to the double-strand structure of the DNA, the reads come from either one strand or its complement in reverse order, and the sequencing technologies cannot assure that two reads were sequenced from the same strand. Thus, each read must be considered in two orientations: the one given by the technologies (defined as the *forward* orientation), and its reverse-complement (defined as the *reverse* orientation) (e.g. $ATGCCA$ and $TGGCAT$ are each other’s reverse-complement).

From the reads, genome assembly methods aim to find the longest true DNA sequences. Note that it is sufficient to only find one strand out of two, as the other is obtained by the reverse-complement transformation of the first one. Assembly methods are often split into three major stages: (i) *assembly* of reads based on their overlaps to obtain longer sequences (*contigs*); (ii) *scaffolding*, that aims to obtain an order of oriented contigs, potentially separated by nucleotide distances (*scaffolds*); (iii) *gap-filling*, that aims to complete the assembly by filling the gaps between the contigs in the scaffolds. Here we focus on the scaffolding step uniquely.

The vast majority of proposed scaffolding formulations are based on distances data between the reads (paired-end or mate-pair data) that are adapted for the contigs to obtain scaffolds. Read distances can either be counted to represent a confidence linking information between two contigs [5, 14], either be considered precisely for contig nucleotide positioning [1], or combined both approaches as in [12, 18, 21].

### Chloroplast genome architecture and structural haplotypes

**Fig. 1:**
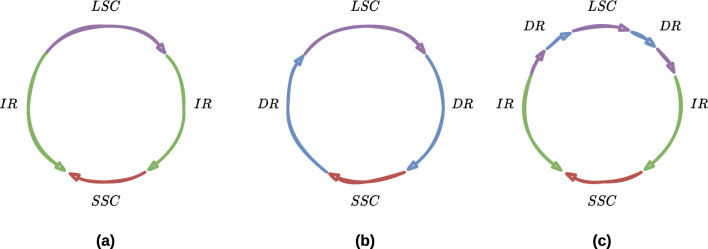
The most studied chloroplast genome form is circular and very often quadripartite. For each of the three figures, coloured arrows represent nucleotide sequences. $LSC$ and $SSC$ stand for long and short single copies (purple and red), respectively. They correspond to regions (subsequences) that are not repeated in the genome. On the opposite, $IR$ and $DR$ stand for inverted and direct repeat (green and blue), respectively. **a** This architecture is the most common one and is defined as a quadripartite architecture. The two green $IR$ arrows face each other and illustrates that one is the reverse-complement sequence of the other; **b** the two blue $DR$ arrows are in the same direction that illustrates both have the same nucleotide sequence; **c** the two types of repeat can simultaneously exist in the chloroplast genome, and DRs are shorter than IRs

In this paper, we address the scaffolding problem for the particular class of chloroplast genomes. Chloroplasts are plants’ organelles derived from the integration of a cyanobacterium in an eukaryotic host. They conduct photosynthesis, a process to convert light energy into chemical energy. Over the evolution time, the chloroplast genome has reduced in length and loosed in terms of complexity [22]. As a result, chloroplast genomes possess few repeats that are usually identical in nucleotide sequences. One the most studied forms of chloroplast genome is a circular quadripartite DNA molecule. It consists of four *regions*: two identical (or highly similar) nucleotide subsequences, separated by two long and short single-copies (*LSC*, *SSC*) [4, 17]. There are two types of repeats: (i) the *Direct Repeat (DR)*, where the sequences are highly similar; (ii) the *Inverted Repeat (IR)*, where one sequence is the reverse-complement of the other. Figure 1 illustrates the common chloroplast genome architectures.

Furthermore, each chloroplast has multiple copies of its genome, and the molecular forms of the copies differ (*structural haplotype* leading to heteroplasmy, and multigenomic structures—not discussed here [3, 16]). This phenomenon can be induced by *flip-flop inversion*: one subsequence is reverse-complemented (*reversed*) during the DNA replication. This inversion is provoked by the existence of facing IR on either side of the reversed subsequence.

**Fig. 2:**
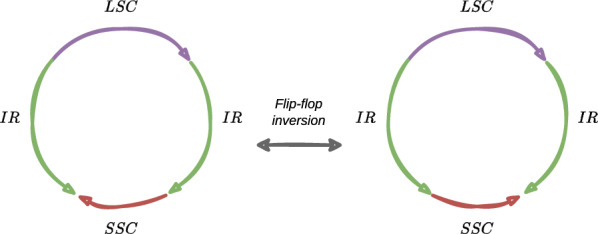
During the DNA replication of the chloroplast genome, one of the region between the two inverted repeats can be reversed (c.f. the red region $SSC$). This provokes the existence of several forms of the genome in the same chloroplast (heteroplasmy)

### Chloroplast scaffolding approaches

Although there are chloroplast genome assemblers and scaffolders, they do not fully exploit the chloroplast genome’s specificities. Some of them are pipelines of generic methods applied on cleaned input dataset [2], or based on locally approaches as seed-and-extend algorithms [7, 8]. Ref. [13], in GetOrganelle, statistically compute the contigs’ multiplicities by minimising the squared distance between them and the mapping coverage by the reads.

Concerning the handling of the distinct genome forms, GetOrganelle returns several solutions and explores in post-process the corresponding architectures. In [1] the flip-flop inversion breakpoints are detected in a post-scaffolding-process, and new optimal solutions can be constructed in polynomial time.

We raise the following two questions: (i) how to mathematically model chloroplast genomic biological knowledge? (ii) How to include the multimerism into the scaffolding problem formulation?

To formalise the scaffolding by integrating the structural haplotypes in its core, we postulate on the particularities of the chloroplast genome: (i) repeats are pairs of regions; (ii) the two regions of a repeat have identical (or reversed) nucleotide sequence; (iii) structural haplotypes can be seen as permutations in a sequence of oriented contigs.

### Organisation of the paper

We first describe the input data for our approach and provide mathematical definitions (Section "Input data and notation"). Based on the above three assumptions, we propose a new formulation for scaffolding chloroplast genomes without requiring any distances (Section "Chloroplast scaffolding problem formulations"). Our approach is region-driven and focuses on retrieving the repeats first. We model the optimisation problem on a directed graph (digraph) where we apply several integer linear programming (ILP) strategies (Sections "Graph and repeated fragment sets" and "Integer linear programming (ILP) formulation"). We then detail how we combine the ILP solutions (Section "Hierarchical problem succession").

The ILP’s solutions and the digraph correspond to an oriented contig sequence representing one genome’s form (Section "From an ILP solution to a genome structure"). We partition this sequence into genomic regions we express in a region graph. This graph enables us to return multiple genome forms (Section "Multiple genome forms").

We prove the decision version of the chloroplast genome scaffolding problem to be $\mathcal{N}\mathcal{P}$-Complete (Section "[Sec Sec19]"). However, we exactly solve the problem by profiting from the small size of the chloroplast instances and providing some numerical results (Section "Numerical results"). We finally conclude (Sections "Conclusion" and "Discussion and perspectives").

## Input data and notation

**Table 1:** Toy example of input data

Contig set			Link set			
Contig	Mult	Wex	Contig	Orient	Contig	Orient
$a$	1	0.70	$a$	$f$	$c$	$r$
$b$	2	0.83	$a$	$r$	$c$	$r$
$c$	2	0.17	$b$	$r$	$c$	$f$
$d$	1	0.43	$b$	$f$	$d$	$f$
			$b$	$f$	$d$	$r$

Left: set of contigs $\mathcal {C}{}$. Right: set of links $\mathcal {L}{}$. For the sake of space, for no one of the links in the table, its reverse is given, although it belongs to $\mathcal {L}{}$

### Set of contigs $\mathcal {C}$

Contigs are words in the nucleotides DNA alphabet $\Sigma _{nuc}{}^+$.

A contig can occur in the genome up to an integer called *multiplicity*. Function $mult:\mathcal {C}{} \rightarrow \mathbb {N}{}_{>0}$ provides its value. Any of contig’s *occurrence* can appear in one of two possible reverse-complementary and mutually exclusive *orientations*: *forward* ($f = 0$) and *reverse* ($r = 1$).

Each contig is provided with an *existence-weight* in $\mathbb {R}{}_{\ge 0}$ given by the function $wex$. The weight is proportional to the number of times a contig aligns with chloroplast sequences from a given set (from related or unrelated species).

Finally, one contig in this set is defined as the *starter* ($s$) that must uniquely participate in the genome ($mult(s) = 1$). The starter is a contig whose sequence matches a sequence shared by most chloroplast genomes in a single-copy.

Table 1 gives an example of contig set.

### Set of links $\mathcal {L}$

Each link is an ordered pair of oriented contigs. We denote by $\mathcal {L}{} \subset \left( {\mathcal {C}{} \times \{f, r\}{}}\right) ^2$ the link set. The nature of the double-strands DNA requires that $\forall (c, d) \in \mathcal {L}{}, (\overline{d}, \overline{c}) \in \mathcal {L}{}$, where $\overline{c}$ and $\overline{d}$ denote the oriented contigs $c$ and $d$ in their reverse orientation, respectively (note that $\overline{\overline{c}} = c$). The links between two oriented contigs $c$ and $d$ are valid for all occurrences of $c$ and $d$ respecting the same orientations. Table 1 gives an example of link set.

### Mathematically defining genomic regions

We aim to order oriented occurrences of the contigs based on their links. Each genome form corresponds to a sequence of oriented contigs. Not all contigs or their occurrences are included. Indeed, the contig set may contain contigs belonging to the plant genome or other organelles. The link set may also contain artefact links. Definition 1 provides the properties the sequence of oriented contigs must respect.

#### Definition 1

(Sequence of oriented contigs) Let $SOC = \left( {c_0, c_1, \dots , c_{n-1}}\right)$ be a sequence of oriented contigs:

- $$\forall i \in \llbracket {0,n-1}\llbracket , (c_i, c_{i+1}) \in \mathcal {L}{}$$;
- $$\forall c \in \mathcal {C}{}, \sum _{c_i \in SOC \mid c_i = c} 1 \le mult(c)$$.

Based on the biological knowledge, we address the dedicated chloroplast scaffolding problem as a region-driven scaffolding, such that specific regions fit into a circular structure. We identify three types of regions to scaffold: the directed repeats, the inverted repeats and the single-copies.

#### Definition 2

(Region) A region $r = \left( {c_0, c_1, \dots , c_{n-1}}\right)$ is a sequence of oriented contigs. Each region is oriented. Let $\overline{r} = \left( {\overline{c_{n-1}}, \ldots , \overline{c_1}, \overline{c_0}}\right)$ be the reverse region of $r$. It is composed of the oriented contigs of $r$, considered in their reverse orientation, and given in the reversed order. According to the reverse symmetry in the links, $\overline{r}$ also respects Definition 1.

#### Definition 3

(Direct repeat—DR) A DR is a couple of regions $(dr_i, dr_j)$ where $dr_i = dr_j$.

#### Definition 4

(Inverted repeat—IR) An IR is a couple of regions $(ir_k, ir_l)$ where $ir_l = \overline{ir_k}$.

#### Definition 5

(Repeat) A repeat is the generic term to denote either DR or IR. The length $replen(R)$ of a repeat $R = (r_i, r_j)$ equals the lengths of $r_i$ and $r_j$ ($replen(R) = |{r_i}| + |{r_j}|$).

#### Definition 6

A SC is a region that is not part of a repeat.

#### Definition 7

(Region weight) The weight $rwex(r)$ of a region $r$ is defined as $rwex(r) = \sum _{c \in r} wex(c)$.

A chloroplast genome consists of a sequence of oriented regions. A genome form is a result of iterative transformations of an initial one. Section "Multiple genome forms" introduces the region graph to model multiple genome forms (sequences of oriented contigs).

#### Definition 8

(Sequence of oriented regions) Consider a sequence $SOR = \left( {r_0, r_1, \dots , r_{n-1}}\right)$:

- $$\forall i \in \llbracket {0,n-1}\llbracket , \left( {r_i[|{r_i}|-1], r_{i+1}[0]}\right) \in \mathcal {L}{}$$^1^
- $$\forall c \in \mathcal {C}{}, \sum _{r \in SOR}\sum _{c_i \in r \mid c_i = c} 1 \le mult(c)$$.

## Chloroplast scaffolding problem formulations

Solving the repeats is the most challenging task. A formulation that does not restrict the occurrences can lead to misassemblies where the results are longer than the solution genomes. Therefore, the use of an occurrence is limited to conformity with the biological knowledge of genome forms. A contig should participate in the sequence only if it enables the formation of pairs of repeated regions. In this case, we would be inclined to assemble the minimum number of repeats.

**Fig. 3:**
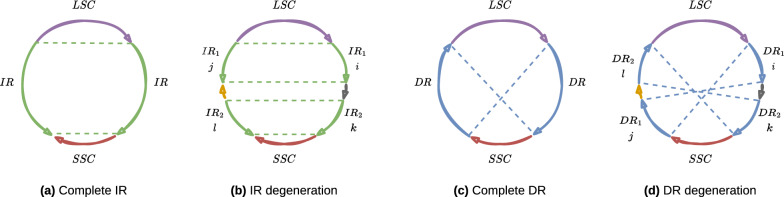
With time, a repeat may degrade so that its occurrences differ. **a** and **c** show two common quadripartite structures with an IR and a DR, respectively. **b** and **d** highlight the impact of a degeneration on their structures. We give the bellow region orders according to the LSC arrow’s direction. In **b**, the degeneration results in two IRs: $IR_1 = (i, j)$ and $IR_2 = (k, l)$, such that $i$ is before $k$ and $l$ precedes $j$. In **d** it results in two DRs: $DR_1 = (i, j)$ and $IR_2 = (k, l)$, such that $i$ is before $k$ and $j$ precedes $l$

However, in the case of repeat degeneration (e.g. two subsequences inside the two regions of an identified repeat differ, note that some IR losses have been reported in the chloroplast genomes of green algae—[20]) finding the minimum number of repeats is not an appropriate model. Figure 3 illustrates the impact of degenerations on quadripartite structures. Indeed, in Fig. 3b, we cannot guaranty to find both $IR_1$ and $IR_2$, but perhaps only one of them. For each repeat type, we address this issue by maximising the cumulative repeat lengths only if their regions respect a specific order.

### Definition 9

(Chloroplast scaffolding problem $\mathcal {CHSP}$) Given a set of contigs with their multiplicities and their weights, a starting contig and a link set. The aim is to obtain a circular sequence of oriented regions maximising the cumulative repeat lengths and minimising the number of repeats, with single-copies of maximum-weight.

For instance, let (A) and (B) be two distinct and feasible sequences of oriented contigs:

(A) $(\dots , a, b, c, d, \dots , a, b, c, d, \dots )$ has one DR $(i, j)$, where $i = j = (a, b, c, d)$;
(B) $(\dots , a, b, \dots , c, d, \dots , a, b, \dots , c, d, \dots )$ has two DRs $(k, l)$ and $(m, n)$, where $k = l = (a, b)$ and $m = n = (c, d)$.

cFor (A) and (B) the cumulative lengths are the same ($replen((i, j)) = replen((k,l)) + replen((m, n)) = 8$). However, (A) has one less repeat, which we prefer.

$\mathcal {CHSP}$ involves three subproblems, each one associated with a particular type of region: (i) $\mathcal {DRP}$ for the direct repeats (Definition 10); (ii) $\mathcal {IRP}$ for the inverted repeats (Definition 11) and (iii) $\mathcal {SCP}$ for the single-copies (Definition 12). We tackle $\mathcal {CHSP}$ in a hierarchical succession of $\mathcal {DRP}$, $\mathcal {IRP}$ and $\mathcal {SCP}$ (Definition 14). Any intermediate problem must preserve the regions found by its predecessors (Definition 13).

$\mathcal {DRP}$ and $\mathcal {IRP}$ constrain the number of occurrences to the structure of pairs of repetitions. Indeed, each repeat type defines a valid repeat structure. The problems consist in maximising the cumulative length of the minimum number of repeats.

### Definition 10

(Chloroplast direct repeat scaffolding problem $\mathcal {DRP}$) Consider a set of contigs, their multiplicities, a starting contig and a link set. Find a circular sequence of oriented regions $SOR$, such that:

- It maximises the cumulative length of the minimum number of DRs, joined by regions of any kind;
- For any couple of DRs $(i, j)$ and $(k, l)$ found in $SOR$ such that their respective positions in $SOR$ given by function $\sigma$ respect $\sigma (i) < \sigma (j)$, $\sigma (k) < \sigma (l)$, and $\sigma (i) < \sigma (k)$, then:
  - $$\left[\kern-0.15em\left[ {\sigma (i),\,\sigma (j)} \right]\kern-0.15em\right] \cap {\text{ }}\left[\kern-0.15em\left[ {\sigma (k),\,\sigma (l)} \right]\kern-0.15em\right] = \varnothing$$;
  - or $$\left[\kern-0.15em\left[ {\sigma (k),\,\,\sigma (j)} \right]\kern-0.15em\right] \subset \left[\kern-0.15em\left[ {\sigma (i),\,\,\sigma (l)} \right]\kern-0.15em\right]$$.

### Definition 11

(Chloroplast inverted repeat scaffolding problem $\mathcal {IRP}$) Consider a set of contigs, their multiplicities, a starting contig and a link set. Find a circular sequence of oriented regions $SOR$, such that:

- It maximises the cumulative length of the minimum number of IRs, joined by regions of any kind;
- For any couple of IRs $(i, j)$ and $(k, l)$ found in $SOR$ such that that their respective positions in $SOR$ given by function $\sigma$ respect $\sigma (i) < \sigma (j)$, $\sigma (k) < \sigma (l)$ and $\sigma (i) < \sigma (k)$, then:
  - $$\left[\kern-0.15em\left[ {\sigma (i),\,\sigma (j)} \right]\kern-0.15em\right] \cap {\text{ }}\left[\kern-0.15em\left[ {\sigma (k),\,\sigma (l)} \right]\kern-0.15em\right] = \varnothing$$;
  - or $$\left[\kern-0.15em\left[ {\sigma (k),\,\,\sigma (l)} \right]\kern-0.15em\right] \subset \left[\kern-0.15em\left[ {\sigma (i),\,\,\sigma (l)} \right]\kern-0.15em\right]$$.

**Fig. 4:**
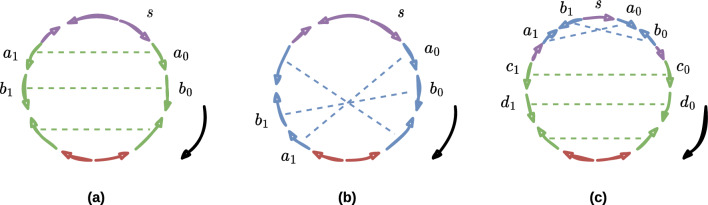
Chloroplast repeat scaffolding. Each subfigure is a common chloroplast genome structure with its associated order of oriented contigs (coloured arrows). The green and the blue sequences of arrows are IR and DR, respectively. The purple and the red ones are single-copy regions. Contig $s$ is the starter, and the right side black arrow determines the contigs’ order. Contigs $a_0$, $a_1$, $b_0$, $b_1$, $c_0$, $c_1$ and $d_0$, $d_1$ are two occurrences of four contigs $a$, $b$, $c$ and $d$, respectively. Each coloured dashed line links two occurrences of the same contig. **a** The order of the occurrences is reversed, and their arrows are oppositely oriented. Visually, an IR produces parallel dashed lines. **b** The order and the orientation of the occurrences is preserved, revealing a DR. **c** A chloroplast genome can contain the two types of repeats. Here, we will retain the hierarchical problem succession $\mathcal {IRP}$–$\mathcal {DRP}$–$\mathcal {SCP}$ ($h_2$) since the IR contains more contigs than the DR

Figure 4 provides examples of valid oriented contig positioning for each common genome structure (Fig. 1). Although Fig. 8 illustrates the authorised and forbidden positions for the latter defined repeated fragments, it is also applicable for the $\mathcal {DRP}$ and $\mathcal {IRP}$ regions’ position cases.

### Definition 12

(Chloroplast single-copy scaffolding problem $\mathcal {SCP}$) Consider a set of contigs, their multiplicities, their weights, a starting contig and a link set. Find a circular sequence of oriented regions such that all the single-copies maximise their weights.

Note that in Definition 12, if they are no repeats, the problem is reduced to find the maximum-weighted circuit of oriented contigs.

### Definition 13

(Chloroplast scaffolding problem succession) $\mathcal {DRP}$, $\mathcal {IRP}$ and $\mathcal {SCP}$ (Definitions 10, 11, 12) can also take as input a set of fixed regions that must be preserved in the resulting sequence of oriented regions.

Our hierarchical approach prioritises the scaffolding of the repeats previously to the SC regions. Indeed, scaffolding the repeats is the most difficult task as it can lead to misassemblies (wrongly chosen links). Hence, a contig should only be used as many times as possible if its occurrences enable the scaffolding of repeats that most closely represent the architecture of the chloroplast genome, as illustrated in Figs. 1 and 4. We assume that the weights concern events that are less relevant comparing to the genomes architecture. Also, the weights on the contigs are less relevant than if they were weights on the links.

### Definition 14

(Hierarchical problem succession) The form of each solution of $\mathcal {CHSP}$ satisfies one of the two problem successions: $\mathcal {DRP}$–$\mathcal {IRP}$–$\mathcal {SCP}$ ($h_1$) and $\mathcal {IRP}$–$\mathcal {DRP}$–$\mathcal {SCP}$ ($h_2$).

The next question is how to prioritise $\mathcal {DRP}$ and $\mathcal {IRP}$? We propose resolving the order by comparing the scores defined in Sect. "Scaffolding problems ILP": if $\mathcal {DRP}$ score is better than this of $\mathcal {IRP}$, then the retained succession will be $\mathcal {DRP}$–$\mathcal {IRP}$–$\mathcal {SCP}$, otherwise it will be $\mathcal {IRP}$–$\mathcal {DRP}$–$\mathcal {SCP}$. In the equality case, we discriminate at a further step of the hierarchical successions. The process is detailed in Sect. "Hierarchical problem succession".

Finally, each hierarchical problem succession produces a circular sequence of oriented regions. From the obtained sequence it is possible to extract a set of ordered pairs of oriented regions. This procedure allows the building of several circular sequences of oriented regions of the same length. Each of them represents one possible chloroplast genome form. This all-equivalent-form process is described in Sect. "Multiple genome forms".

## Graph and repeated fragment sets

In order to efficiently handle the multiplicities of the contigs, hence of the links, we need to build adapted data structures. On the one hand, finding a sequence of oriented contigs, when the links correspond to ordered pairs of oriented contigs, justifies the use of a directed graph to represent the oriented contigs and their links. Section "Graph structure" defines such a directed graph structure. On the other hand, scaffolding the repeats requires choosing pairs of contigs occurring several times in the oriented contig sequence. Section "Repeated fragment sets" defines the sets of such repeat contig candidates.

### Graph structure

Here we describe a directed graph suitable for further algorithms and the mathematical formulation of the scaffolding problems from Definitions 10, 11, 12.

#### Definition 15

(Multiplied Doubled Contig Graph—$MDCG{}$) Given a set of contigs $\mathcal {C}{}$, their multiplicities and the link set $\mathcal {L}{}$, the multiplied doubled contig graph $MDCG{} = (V, E, vwex)$ is defined such that:

$$\begin{aligned} V = \left\{ \begin{aligned}{}&v_{f,0}, \dots , v_{f,n-1}, \\&v_{r,0}, \dots , v_{r,n-1} \end{aligned} \left| \begin{aligned}{}&c \in \mathcal {C}{} \\&n = mult(c) \end{aligned}\right. \right\} \end{aligned}$$

is the set of all the forward an reverse occurrences of all the contigs ($|{V}| = 2 \sum _{c \in \mathcal {C}{}} mult(c)$). The vertices are associated with four functions:

- $contig\colon V \rightarrow{\!\!\!\!\!\!\!\!}\rightarrow \mathcal {C}{}$ provides the contig associated with a vertex;
- $vor\colon V \rightarrow{\!\!\!\!\!\!\!\!}\rightarrow \{f, r\}{}$ provides the orientation of the contig;
- $vocc \colon V \rightarrow \mathbb {N}{}_{>0}$ provides the occurrence number of the contig;
- $vwex \colon V \rightarrow \mathbb {R}{}_{\ge 0}$ provides the weight of each vertex such that $\forall v \in V, vwex(v) = wex(contig(v))$.

$$\begin{aligned} E = \left\{ \begin{aligned}{}&\left( {u, v}\right) \in V^2 \text { s.t. } \\&\begin{pmatrix} contig(u) &{} vor(u) \\ contig(v) &{} vor(v) \end{pmatrix} \in \mathcal {L}{} \end{aligned} \right\} \end{aligned}$$

is the set of multiplied links ($|{E}| = \sum _{\left( {c, d}\right) \in \mathcal {L}{}} mult(c)mult(d)$).

**Fig. 5:**
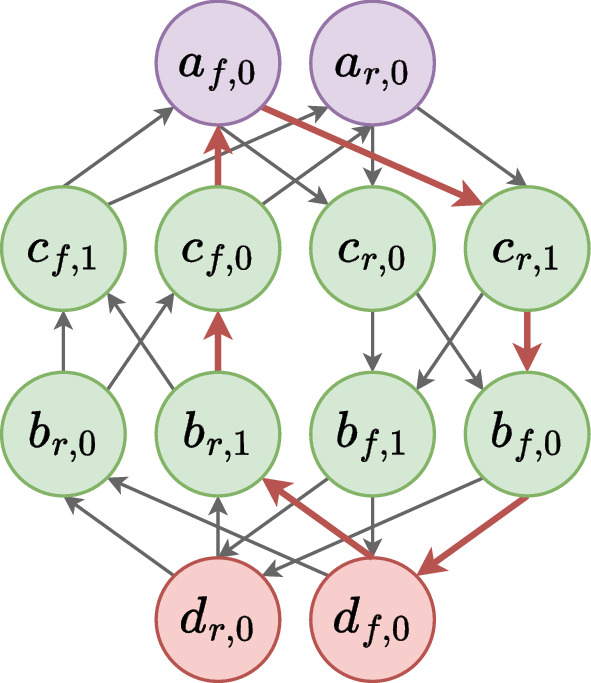
$MDCG$ example. Each vertex is associated with an occurrence of an oriented contig, and each contig is represented by an even number of vertices. For example, vertex labelled $c_{r,1}$ means that it comes from contig $c = contig(c_{r,1})$, in its reverse orientation ($vor(c_{r,1}) = r$), and in its second occurrence ($vocc(c_{r,1}) = 1$). The colours are the same as the ones in Fig. 1a to relate the input data with the IR architecture. The bold red edges draw a circuit corresponding to an IR scaffolding where $a_{f,0}$ is the starter

Figure 5 illustrates the $MDCG$ representing the example data given in Table 1.

### Repeated fragment sets

A repeat is a couple containing two identical (or reverse for IR) sequences of oriented contigs (Definitions 3 and 4). Therefore, a repeat consists of couples containing two identical (or reverse) contigs. In the context of $MDCG$, this leads to the concept of *repeated fragments*.

#### Definition 16

A repeated fragment is an unordered pair of vertices such that one of the corresponding oriented contig belongs to the first region of a repeat, while the other belongs to the second region. The vertices are associated with the same contig but their occurrences differ, i.e. for each repeat $(r_i, r_j)$, $\exists u, v \in V, c \in \mathcal {C}{}$ where $contig(u) = contig(v) = c$ and $vocc(u) \ne vocc(v)$ such that $(c, vor(u)) \in r_i$ and $(c, vor(v)) \in r_j$.

**Fig. 6:**
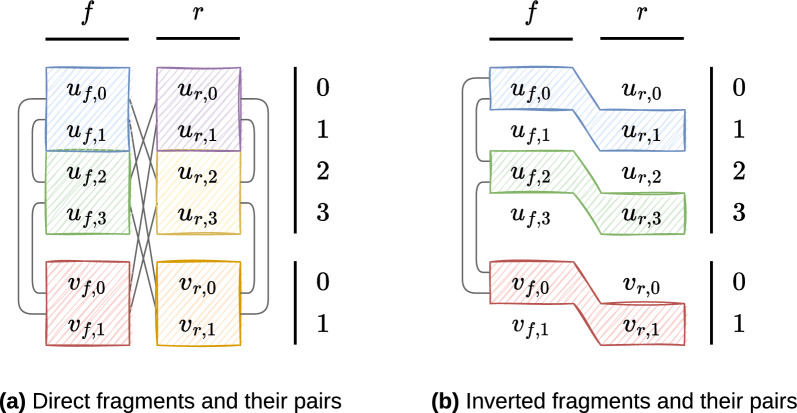
Repeated fragment sets illustration for two contigs $c$ and $d$. In the two subfigures, $mult(c) = 4$ and $mult(d) = 2$, so that $contig(u_{or,occ}) = c$ and $contig(v_{or,occ}) = d$. Two vertices coming from the same contig are respectively direct/inverted fragments if they are in the same coloured box, and so they belong to $DirF{}$/$InvF{}$. A tight grey line connects two direct/inverted fragments if their pair belong to $PDirF{}$/$PInvF{}$. **a** $|{DirF{}}| = 6$, and, e.g. $(u_{f,0}, u_{f,1}) \in DirF{}$ so $dirfrag\,({u_{f,0}}) = dirfrag\,({u_{f,1}}) = (u_{f,0}, u_{f,1})$. $|{PDirF{}}| = 12$, and, e.g. $((u_{f,0}, u_{f,1}), (u_{r,2}, u_{r,3})) \in PDirF{}$. **b** $|{InvF{}}| = 3$, and, e.g. $(u_{f,2}, u_{r,3}) \in InvF{}$ so $invfrag\,({u_{f,2}}) = invfrag\,({u_{r,3}}) = (u_{f,2}, u_{r,3})$. $|{PInvF{}}| = 3$, and, e.g. $((u_{f,2}, u_{r,3}), (v_{f,0}, v_{r,1})) \in PInvF{}$

**Fig. 7:**
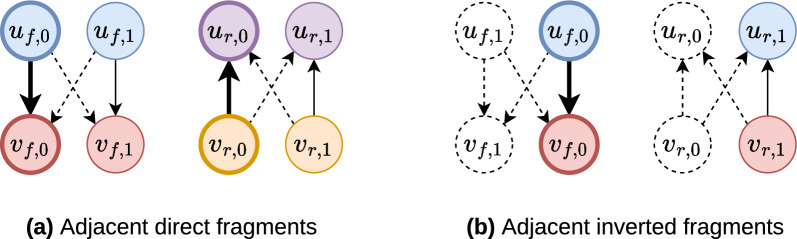
Adjacent repeated fragment sets examples. The two subfigures represent the multiplied link (and its reverse) $((c, f), (d, f)) \in \mathcal {L}{}$, where $mult(c) = 2$ and $mult(d) = 2$, so that $contig(u_{or,occ}) = c$ and $contig(v_{or,occ}) = d$. Two vertices of the same colour visualise a repeated fragment. Bold edges (canonical) are the ones that belong to the adjacent repeated fragments sets. The functions $diradj$/$invadj$ enable to retrieve the normal edges with the bold ones, and vice-versa. Dashed edges do not participate in $ADirF{}$/$AInvF{}$. Remember that $\forall (u, v) \in E, \left( {\overline{v}, \overline{u}}\right) \in E$. **a** $diradj\,({u_{f,0}, v_{f,0}}) = \left( {u_{f,1}, v_{f,1}}\right)$; **b** $invadj\,({u_{f,0}, v_{f,0}}) = \left( {v_{r,1}, u_{r,1}}\right)$

For example, $(c_{f,0}, c_{r,1})$ is a repeated fragment for the IR in Fig. 5. We then precise the set of repeated fragments for each repeat type. Denote by $\mathcal {R}{} = \left\{ c \in \mathcal {C}{} \mid mult(c) > 1 \right\}$ the set of contigs candidate to be part of repeats. For the sake of clarity, for each vertex $v \in V$, we note $ctg_{v} = contig(v)$, $or_v = vor(v)$, $occ_v = vocc(v)$, $wex_v = vwex(v)$ and $mult_v = mult(contig(v))$. We also assume there is an arbitrary strict total order on $\mathcal {C}$, i.e. $\forall c, d \in \mathcal {C}{}, c \ne d \iff c < d \veebar c > d$.

#### Definition 17

A direct fragment $(u, v) \in V^2$ is a repeated fragment, such that $u$ and $v$ have the same orientation.

$$\begin{aligned} DirF{} = \bigcup _{c \in \mathcal {R}{}} \left\{ \begin{aligned}{}&(i, j) \in V^2 \text { s.t.} \\&\quad ctg_i = ctg_j = c \\&\quad \wedge or_i = or_j \in \{f, r\}{} \\&\quad \wedge occ_i = occ_j - 1 = 2 k\\&\quad 0 \le k < \left\lfloor {\frac{mult(c)}{2}}\right\rfloor \end{aligned}\right\} \end{aligned}$$

#### Definition 18

An inverted fragment $(u, v) \in V^2$ is a repeated fragment, such that the orientations of $u$ and $v$ differ.

$$\begin{aligned} InvF{} = \bigcup _{c \in \mathcal {R}{}} \left\{ \begin{aligned}{}&(i, j) \in V^2 \text { s.t.} \\&\quad ctg_i = ctg_j = c \\&\quad \wedge or_i = f \wedge or_j = r \\&\quad \wedge occ_i = occ_j - 1 = 2 k\\&\quad 0 \le k < \left\lfloor {\frac{mult(c)}{2}}\right\rfloor \end{aligned}\right\} \end{aligned}$$

Figure 6 illustrates $DirF{}$ and $InvF{}$ sets. In addition, we add two functions to retrieve the repeated fragments from the vertices in $\Theta {}(1)$: $dirfrag \colon V' \subset V \rightarrow DirF{}$ and $invfrag\colon V' \subset V \rightarrow InvF{}$ (abstracted with the *repfrag* function, c.f. Appendix A for their definitions).

Furthermore, Definitions 10 and 11 constrain the order between the repeated fragments. Hence, they respectively require to compare pairs of direct/inverted fragments, that must be defined:

#### Definition 19

(Set of pairs of direct fragments)

$$\begin{aligned} PDirF{} = \left\{ \begin{aligned}{}&\big ((i, j), (k, l)\big ) \in DirF{}^2 \text { s.t.} \\&\quad ctg_j< ctg_k \\&\quad \vee \\&\quad ctg_j = ctg_k \wedge occ_j < occ_k \end{aligned}\right\} \end{aligned}$$

#### Definition 20

(Set of pairs of inverted fragments)

$$\begin{aligned} PInvF{} = \left\{ \begin{aligned}{}&\big ((i, j), (k, l)\big ) \in InvF{}^2 \text { s.t.} \\&\quad ctg_j< ctg_k \\&\quad \vee \\&\quad ctg_j = ctg_k \wedge occ_j < occ_k \end{aligned}\right\} \end{aligned}$$

Figure 6 illustrates $PDirF{}$ and $PInvF{}$ sets. The constraints defining $DirF$, $InvF$, $ADirF$, $AInvF$, $PDirF$ and $PInvF$ are explained in details in Appendix B where we proof they are the smallest sets enabling to find all the distinct solutions.

Furthermore, a repeat is a couple of regions (Definition 5), themselves defined as oriented contig sequences (Definition 2). We need to define the edges connecting two repeated fragments.

#### Definition 21

An adjacent repeated fragment is an edge $(u, v) \in E$ such that $u$ and $v$ participate in two distinct repeated fragments.

#### Definition 22

An adjacent direct fragment is an edge between two direct fragments. Let $ADirF{}$ be the set of adjacent direct fragments:

$$\begin{aligned} ADirF{}= & {} {}&\left\{ \begin{aligned}{}&(u, v) \in E \text { s.t.} \\&\quad ctg_u \ne ctg_v \\&\quad \wedge occ_u = 2 k \\&\quad 0 \le k< \left\lfloor {\frac{mult_u}{2}}\right\rfloor \\&\quad \wedge occ_v = 2 k'\\&\quad 0 \le k'< \left\lfloor {\frac{mult_v}{2}}\right\rfloor \\ \end{aligned} \right\} \\&\bigcup{} & {} \left\{ \begin{aligned}{}&(u, v) \in E \text { s.t.} \\&\quad ctg_u = ctg_v \\&\quad \wedge (or_u = f \vee or_v = f)\\&\quad \wedge occ_u = 2 k\\&\quad \wedge occ_v = 2 k' \\&\quad 0 \le k< k' < \left\lfloor \frac{mult_u}{2}\right\rfloor \end{aligned} \right\} \end{aligned}$$

#### Definition 23

An adjacent inverted fragment is an edge between two inverted fragments. Let $AInvF{}$ be the set of adjacent inverted fragments:

$$\begin{aligned} AInvF{}= & {} {}&\left\{ \begin{aligned}{}&(u, v) \in E \text { s.t.}\\&\quad ctg_u< ctg_v \\&\quad \wedge occ_u = 2 k + or_u\\&\quad 0 \le k< \left\lfloor {\frac{mult_u}{2}}\right\rfloor \\&\quad \wedge occ_v = 2 k' + or_v \\&\quad 0 \le k'< \left\lfloor {\frac{mult_v}{2}}\right\rfloor \\ \end{aligned} \right\} \\&\bigcup{} & {} \left\{ \begin{aligned}{}&(u, v) \in E \text { s.t.} \\&\quad ctg_u = ctg_v \\&\quad \wedge (or_u = f \vee or_v = f)\\&\quad \wedge occ_u - or_u = 2 k \\&\quad \wedge occ_v - or_v = 2 k'\\&\quad 0 \le k< k' < \left\lfloor \frac{mult_u}{2}\right\rfloor \end{aligned}\right\} \end{aligned}$$

Edges in $ADirF$ and $AInvF$ play the role of *canonical edges* between two adjacent repeated fragments, see Fig. 7. In addition, we add two functions to retrieve the adjacent repeated fragments from the edges in $\Theta {}(1)$: $diradj\colon E \rightarrow E$ and $invadj\colon E \rightarrow E$ (abstracted with the *repadj* function, c.f. Appendix A for their definitions).

## Integer linear programming (ILP) formulation

Modelling $\mathcal {DRP}$, $\mathcal {IRP}$ and $\mathcal {SCP}$ from Definitions 10, 11, 12 requires finding a valid circuit in $MDCG$.

### Definition 24

(Valid circuit in $MDCG$) Given a graph $MDCG{} = (V, E)$ and a starting vertex $s$, where $ctg_s$ is the starting contig, $or_s = f$ and $occ_s = 0$. A circuit $cp$ in $MDCG$ is valid if:

- It starts and ends with $s$;
- $$\forall \, v \in cp, \overline{v} \notin cp$$, where $$ctg_v = ctg_{\overline{v}}$$, $$or_v = 1 - or_{\overline{v}}$$ and $$occ_v = occ_{\overline{v}}$$;
- Consecutive vertices $u$ and $v$ in $cp$ are connected by an edge $(u, v) \in E$.

First we describe common constraint blocks in Sects. "Circuit constraints", "Repeated regions constraints" and "Fixing regions constraints" for ILPs formulations, and then we give the $\mathcal {DRP}$, $\mathcal {IRP}$ and $\mathcal {SCP}$ scaffolding problems ILP in Sect. "Scaffolding problems ILP".

Let ${\textsf{M}{}} = \sum _{c \in \mathcal {C}{}} mult(c)$ be a constant, $N^-_v$ and $N^+_v$ be the sets of predecessors and successors of vertex $v \in V$, respectively.

### Circuit constraints

The following set of constraints defines a valid circuit of oriented contig in $MDCG$, and is defined with a flow formulation as in [1] instead of using Miller-Tucker-Zemlin constraints to avoid cycles [15].

### Binary variables

- $x_e$ encodes if the edge $e \in E$ participates in the circuit.

### Continuous variables

- $i_v \in [0, 1]$ encodes if the vertex $v \in V {\setminus } \left\{ s, \overline{s}\right\}$ participates in the circuit. Although it is a continuous variable, it acts as a binary one as proven in [9].
- $f_e \in \mathbb {R}{}_{\ge 0}$ is the positive flow on the participating edge $e \in E$ in the circuit (zero otherwise).

Constraint (1) defines the flow. The circuit must start and end with the starter in its forward orientation (Constraints (2, 3, 4, 5). If a vertex participates, its reverse cannot (Constraint (6)). Defining a circuit is equivalent to requiring an edge to exit a vertex if it has an incoming one (Constraint (7)). Constraint (8) forces the flow to be monotonically increasing. This property avoids cycles.

### $CCircuit$ constraints

1 $$\begin{aligned}{}&x_e \le f_e \le \mathsf{M} x_e{} & {} \forall e \in E \end{aligned}$$

2 $$\begin{aligned}{}&\sum _{v \in N^+_s} x_{sv} = \sum _{v \in N^-_s} x_{vs} = 1{} & {} \end{aligned}$$

3 $$\begin{aligned}{}&\sum _{v \in N^+_s} f_{sv} = 1{} & {} \end{aligned}$$

4 $$\begin{aligned}{}&x_{v\overline{s}} = 0{} & {} \forall v \in N^-_{\overline{s}} \end{aligned}$$

5 $$\begin{aligned}{}&x_{\overline{s}v} = 0{} & {} \forall v \in N^+_{\overline{s}} \end{aligned}$$

6 $$\begin{aligned}{}&\forall v \in V \setminus \left\{ s, \overline{s}\right\} : \nonumber{} & {} \\&\quad i_v + i_{\overline{v}} \le 1{} & {} \end{aligned}$$

7 $$\begin{aligned}{}&\quad \sum _{u \in N^-_v} x_{uv} \le i_v \le \sum _{w \in N^+_v} x_{vw}{} & {} \end{aligned}$$

8 $$\begin{aligned}{}&\quad \sum _{w \in N^+_v} f_{vw} - \sum _{u \in N^-_v} f_{uv} = i_v{} & {} \end{aligned}$$

$$\begin{aligned}{}&x_e \in \left\{ 0, 1\right\} {} \quad \forall e \in E{} & {} \nonumber \\&i_v \in [0, 1] \quad \forall v \in V \setminus \left\{ s, \overline{s}\right\}{} & {} \nonumber \\&f_e \in \mathbb {R}{}_{\ge 0} \quad \forall e \in E{} & {} \end{aligned}$$

### Repeated regions constraints

The following constraints are general to define ILPs for $\mathcal {DRP}$ and $\mathcal {IRP}$. Definitions 10 and 11 define the repeated regions according to the positions of the oriented contig in them. It follows that some order of the vertices in the pairs of repeated fragments are allowed, and some others are forbidden. We decide to write the constraints for the forbidden cases because they are fewer than the allowed ones. To model the forbidden orders between 4 vertices, we compare the positions between two.

Specifically for $\mathcal {IRP}$, modelling the forbidden orders echoes the approach for the RNA folding problem [11], except that the positions of the RNA’s nucleotides are known.

According to Definitions 10 and 11, and given $PDirF$ and $PInvF$ (Definitions 19 and 20), denote by $ForbidDR{}$ and $ForbidIR{}$ the sets of forbidden quartet vertices for the DRs and IRs, respectively:

$$\begin{aligned} ForbidDR{}&= \left\{ \begin{aligned}{}&(i, k, l, j) \\&(k, i, j, l) \end{aligned} \left| \begin{aligned}{}&i, j \in p, i \ne j\\&\wedge k, l \in q, k \ne l \\&\forall (p, q) \in PDirF{} \end{aligned} \right. \right\} \\ ForbidIR{}&= \left\{ \begin{aligned}{}&(i, k, j, l) \\&(k, i, l, j) \end{aligned} \left| \begin{aligned}{}&i, j \in p, i \ne j\\&\wedge k, l \in q, k \ne l \\&\forall (p, q) \in PInvF{} \end{aligned}\right. \right\} \end{aligned}$$

**Fig. 8:**
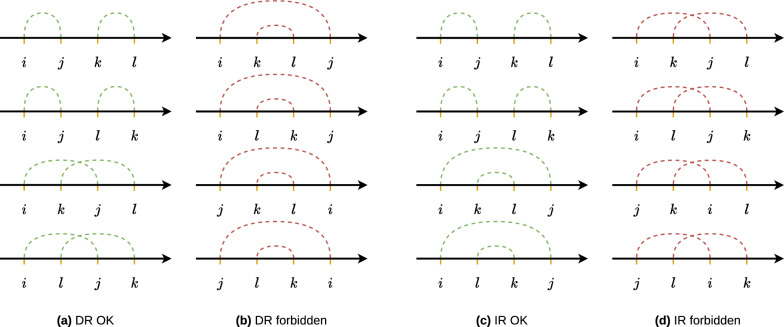
Non-exhaustive illustrations for authorised and forbidden order cases for two repeated fragments $\left( {(i, j), (k, l)}\right) \in PRepF{}$. **a** and **b** Authorised and forbidden orders for $PDirF$; **c** and **d** Authorised and forbidden orders for $PInvF$

Figure 8 illustrates the authorised and forbidden positions for $\mathcal {DRP}$ and $\mathcal {IRP}$.

To know if we are in the forbidden cases described in these two sets, we propose to compare the vertices two-by-two. Denote by $AlphaDR{}$ and $AlphaIR{}$ the sets containing the couples of vertices to be compared to determine the forbidden cases respectively associated with $ForbidDR{}$ and $ForbidIR{}$ sets, such that:

$$\begin{aligned} AlphaDR{}&= \left\{ \begin{aligned}{}&(i, j), (k, l), (i, k), \\&(j, l), (i, l), (j, k)\\&\text {s.t. } \left( {(i, j), (k, l)}\right) \in PDirF{} \end{aligned} \right\} \\ AlphaIR{}&= \left\{ \begin{aligned}{}&(i, k), (i, l), (j, k), (j, l) \\&\text {s.t. } \left( {(i, j), (k, l)}\right) \in PInvF{} \end{aligned} \right\} \end{aligned}$$

In the following, the sets of repeated fragments and these for the forbidden orders are abstracted to generalise $\mathcal {DRP}$ and $\mathcal {IRP}$. Table 2 gives the correspondence of the sets depending on the problem to solve.

**Table 2:** ILP sets and functions corresponding table

ILP	RepF	PRepF	ARepF	
$\mathcal {DRP}$	$DirF$	$PDirF$	$ADirF$	
$\mathcal {IRP}$	$InvF$	$PInvF$	$AInvF$	

ILP	Forbid	Alpha	Repfrag	Repadj
$\mathcal {DRP}$	$ForbidDR$	$AlphaDR$	*dirfrag*	*diradj*
$\mathcal {IRP}$	$ForbidIR$	$AlphaIR$	*invfrag*	*invadj*

### Binary variables

- $m_{ij}$ encodes if the repeated fragment $(i, j) \in RepF{}$ is a part of a repeat.
- $isadj_{e}$ encodes if two repeated fragments connected by the edge $e \in ARepF{}$ (and $repadj(e) \in E$) are adjacent in the circuit.
- $forbid_{ijkl}$ encodes whether we are in the forbidden vertices order $(i, j, k, l) \in Forbid{}$.
- $\alpha _{uv}$ encodes whether the vertex $u$ is before the vertex $v$ in the circuit. Since $\alpha _{uv} = 1 - \alpha _{vu}$, even if $(v, u) \notin Alpha{}$, for clarity we write $\alpha _{vu}$ instead of $1 - \alpha _{uv}$.

### Continuous variables

- $i_v \in [0, 1]$ encodes if the vertex $v \in V {\setminus } \left\{ s, \overline{s}\right\}$ participates in the circuit, and acts as a binary variable.
- $f_e \in \mathbb {R}{}_{\ge 0}$ is the positive flow on the participating edge $e \in E$ in the circuit (zero otherwise). We use the exiting flow to define the position $pos(v)$ of a vertex $v \in V$, i.e. $pos(v) = \sum _{w \in N^+_v} f_w$.

The vertices of participating repeated fragments must be in the circuit (Constraints (9 and 10)). Constraints (11, 12, 13) implement with linear constraints the $\alpha _{uv}$ definition. Constraints (14, 15, 16) implement the $forbid_{ijkl}$ definition. Constraints (17, 18, 19, 20) implement the $isadj_e$ definition.

### $CRepeat$ constraints

9 $$\begin{aligned}{}&\text {Add the set of constraints } CCircuit{}{} & {} \nonumber \\ \nonumber \\&\forall (i, j) \in RepF{}: \nonumber \\&\quad m_{ij} \le i_i{} & {} \end{aligned}$$

10 $$\begin{aligned}{}&\quad m_{ij} \le i_j{} & {} \end{aligned}$$

11 $$\begin{aligned} \nonumber \\&\forall (u, v) \in Alpha{}: \nonumber \\&\quad pos(v) - pos(u) \le \mathsf{M}{} \alpha _{uv}{} & {} \end{aligned}$$

12 $$\begin{aligned}{}&\quad pos(u) - pos(v) \le \mathsf{M}{} (1 - \alpha _{uv}){} & {} \end{aligned}$$

13 $$\begin{aligned}{}&\quad pos(u) + pos(v) \ge \alpha _{uv}{} & {} \end{aligned}$$

14 $$\begin{aligned} \nonumber \\&\forall (i, j, k, l) \in Forbid{}: \nonumber \\&\quad 3 forbid_{ijkl} \le \alpha _{ij} + \alpha _{jk} + \alpha _{kl}{} & {} \end{aligned}$$

15 $$\begin{aligned}{}&\quad 2 + forbid_{ijkl} \ge \alpha _{ij} + \alpha _{jk} + \alpha _{kl}{} & {} \end{aligned}$$

16 $$\begin{aligned} \nonumber \\&\forall (p, q) \in PRepF{}:\nonumber \\&\quad m_p + m_q \le 2 - \sum _{\begin{array}{c} (i, j, k, l)\\ \in Forbid{} \\ \text {s.t. } (p, q) \end{array}} forbid_{ijkl}{} & {} \end{aligned}$$

17 $$\begin{aligned} \nonumber \\&\forall (u, v) \in ARepF{}: \nonumber \\& isadj_{uv} \le x_{uv}{} & {} \end{aligned}$$

18 $$\begin{aligned}{}&\quad isadj_{uv} \le x_{repadj\,({u, v})}{} & {} \end{aligned}$$

19 $$\begin{aligned}{}&\quad isadj_{uv} \le m_{repfrag\,({u})}{} & {} \end{aligned}$$

20 $$\begin{aligned}{}&\quad isadj_{uv} \le m_{repfrag\,({v})}{} & {} \end{aligned}$$

$$\begin{aligned}{}&m_p \in \left\{ 0, 1\right\} {} \quad \forall p \in RepF{} \nonumber \\&isadj_e \in \left\{ 0, 1\right\} {} \quad \forall e \in ARepF{}\nonumber \\&forbid_{ijkl} \in \left\{ 0, 1\right\} {} \quad \forall (i, j, k, l) \in Forbid{} \nonumber \\&\alpha _{uv} \in \left\{ 0, 1\right\} {} \quad \forall (u,v) \in Alpha{} \nonumber \end{aligned}$$

### Fixing regions constraints

When repeats are previously scaffolded, the involved regions are fixed as input for the next problems. Let $ADirF{}^*$, $DirF{}^*$, $AInvF{}^*$ and $InvF{}^*$ respectively be the sets of (adjacent) direct and (adjacent) inverted fragments composing the direct and inverted repeats that have been scaffolded.

### $CFixRegions$ constraints

21 $$\begin{aligned}{}&\forall (u, v) \in DirF{}^* \cup InvF{}^*: \nonumber \\&\quad i_{u} = 1{} & {} \end{aligned}$$

22 $$\begin{aligned}{}& i_{v} = 1{} & {} \end{aligned}$$

23 $$\begin{aligned} \nonumber \\&\forall (u, v) \in ADirF{}^* \cup AInvF{}^*: \nonumber \\&\quad x_{uv} = 1{} & {} \end{aligned}$$

24 $$\begin{aligned} \nonumber \\&x_{diradj\,({u, v})} = 1 \quad \forall (u, v) \in ADirF{}^*{} & {} \end{aligned}$$

25 $$\begin{aligned}{}&x_{invadj\,({u, v})} = 1 \quad \forall (u, v) \in AInvF{}^*{} & {} \end{aligned}$$

### Speed-up constraints

Constraints (26 and 27) prevent the solver to loop on strictly equivalent solutions, e.g. solutions that differ according to a permutation of the occurrences. Denote by $ConsOcc$ the set of occurrence-consecutive vertices, such that:

$$\begin{aligned} ConsOcc{} = \left\{ (u, v) \in V^2 \left| \begin{aligned}{}&ctg_u = ctg_v \\&\wedge or_u = or_v = f\\&\wedge occ_u = occ_v - 1 \end{aligned}\right. \right\} \end{aligned}$$

Also, denote by $ConsRepF$ the set of consecutive repeated fragments, such that:

$$\begin{aligned} ConsRepF{} = \left\{ \begin{aligned}{}&((i, j), (k, l)) \in RepF{}^2 \text { s.t.} \\&\quad ctg_i = ctg_j = ctg_k = ctg_l \\&\quad \wedge or_i = or_k \wedge or_j = or_l\\&\quad \wedge occ_i = occ_k - 2\\&\quad \wedge occ_j = occ_l - 2 \end{aligned} \right\} \end{aligned}$$

26 $$\begin{aligned}{}&i_v + i_{\overline{v}} \le i_u + i_{\overline{u}}{} & {} \forall (u, v) \in ConsOcc{} \end{aligned}$$

27 $$\begin{aligned}{}&m_q \le m_p{} & {} \forall (p, q) \in ConsRepF{} \end{aligned}$$

### Scaffolding problems ILP

Finally, it is possible to define the ILP formulations for the $\mathcal {DRP}$, $\mathcal {IRP}$ and $\mathcal {SCP}$ scaffolding problems as an union of the constraints described before.

For $\mathcal {DRP}$ and $\mathcal {IRP}$ the ILP formulations are the same, and it is sufficient to choose the sets $RepF$, $PRepF$, $ARepF$, $Alpha$, $Forbid$ and $ConsRepF$ according to the repeats the problems scaffold. We aim to maximise the cumulative length of the minimum number of repeats. The objective value corresponds to:

$$\begin{aligned}{}&\quad \sum _{r \in Repeats} replen(r) - |{Repeats}| \\&\quad = \sum _{p \in RepF{}} 2 m_p - \left( {\sum _{p \in RepF{}} m_p - \sum _{e \in ARepF{}} isadj_e}\right) \\&\quad = \sum _{p \in RepF{}} m_p + \sum _{e \in ARepF{}} isadj_e \end{aligned}$$

where $Repeats$ is the set of repeats.

#### $\mathcal {DRP}$ and $\mathcal {IRP}$ models

$$\begin{aligned}{}&{\textsf { max}} \, \sum _{p \in RepF{}} m_p + \sum _{e \in ARepF{}} isadj_e\\&\begin{aligned}{}&\textsf {s.t.}{} & {} CCircuit{}{} & {} \\{} & {} &CRepeat{}{} & {} \\{} & {} &(26){} & {} \\{} & {} &(27){} & {} \textsf{~if~no~repeats~to~fix} \\{} & {} &CFixRegions{}{} & {} \textsf{~otherwise} \end{aligned} \end{aligned}$$

Traditionally, $\mathcal {SCP}$ finds the maximum weighted circuit.

#### $\mathcal {SCP}$ model

$$\begin{aligned}{}&{\textsf { max}} \, \sum _{v \in V \setminus \{s, \overline{s}\}} \mathsf {wex_v} i_v \\&\begin{aligned}{}&\textsf { s.t.}{} & {} CCircuit{}{} & {} \\{} & {} &(26){} & {} \\{} & {} &CFixRegions{}{} & {} \textsf {~if~repeats~to~fix} \end{aligned} \end{aligned}$$

Both for $\mathcal {DRP}$ and $\mathcal {IRP}$, the number of variables and constraints are in $O(|{V}|^2 + |{E}|)$. The number of variables and constraints for $\mathcal {SCP}$ are in $O(|{V}| + |{E}|)$.

## Hierarchical problem succession

Finally, here we describe how we combine the $\mathcal {DRP}$, $\mathcal {IRP}$ and $\mathcal {SCP}$ scaffolding problems. As described in Definition 14, two problem combinations are opposed. The combinations are kept depending on the value of the problems’ objective functions.

### Definition 25

(Hierarchical problem succession solutions) Denote by $h_1, h_2$ the two hierarchical problem successions $\mathcal {DRP}$–$\mathcal {IRP}$–$\mathcal {SCP}$ and $\mathcal {IRP}$–$\mathcal {DRP}$–$\mathcal {SCP}$. For each $h \in \left\{ h_1, h_2\right\}$, denote by $Z^*_h \in \mathbb {R}{}_{\ge 0}^3$ the vector containing the values of the objective functions for each problem in the order of the problem succession corresponding to $h$.

By $S$ we denote the set of optimal problem successions, such that:

$$\begin{aligned} S = \left\{ s \, \left| \, \forall k \in \llbracket {0,2}\rrbracket, Z^*_s[k] = \max _{h \in \left\{ h_1, h_2\right\} } Z^*_h[k] \right. \right\} \end{aligned}$$

Note that $0 \le |{S}| \le 2$, and Definition 25 is stable for any problem with an objective value equal to zero. For example, $Z^*_{h_1}[1] = 0$ means that there is no inverted repeat. For an easier interpretation of the architecture, we adopt a *problem code combination*, summarised in Table 3.

**Table 3:** Problem code combinations

$\textsf{Z}^*_{\textsf{h}}$			$\textsf{h}_{1}$	$\textsf{h}_{2}$
[0]	[1]	[2]	$\mathcal {DRP}$–$\mathcal {IRP}$–$\mathcal {SCP}$	$\mathcal {IRP}$–$\mathcal {DRP}$–$\mathcal {SCP}$
0	0	–	sc	sc
$> 0$	0	–	dr–sc	ir–sc
$> 0$	$> 0$	–	dr–ir–sc	ir–dr–sc

## From an ILP solution to a genome structure

**Fig. 9:**
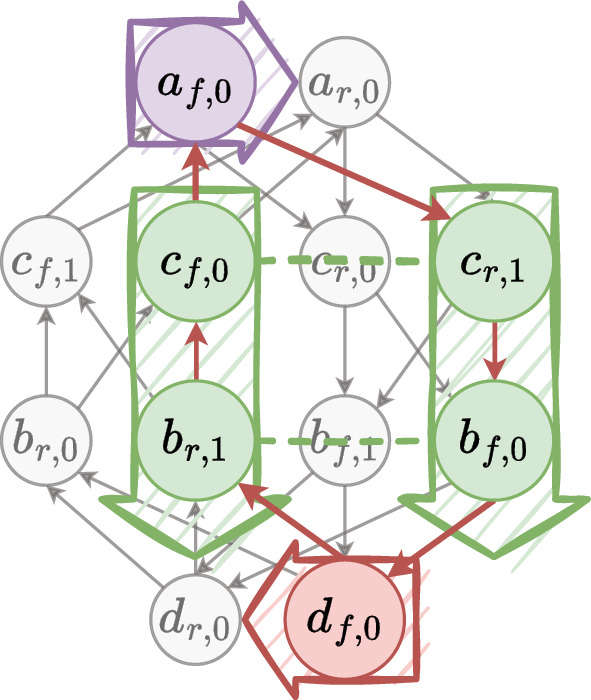
Extracting the genome architecture in $MDCG$ from a $\mathcal {CHSP}$ solution. The illustrated $\mathcal {CHSP}$ solution consists of the red circuit $(a_{f,0}, c_{r,1}, b_{f,0}, d_{f,0}, b_{r,1}, c_{f,0})$ and of the two choosen inverted fragments $InvF{}^* = \left\{ (c_{f,0}, c_{r,1}), (b_{f,0}, b_{r,1})\right\}$ (vertex pairs linked by the green dashed lines). Algorithm 4 returns four regions: $m = 1$ inverted repeat (the two green arrows pointing downwards) and $n = 2$ single-copies (the purple and the red arrows). $COR = \left( {(a_f), (c_r, b_f), (d_f)}\right)$ and $SOR = \left( {0_f, 1_f, 2_f, 1_r}\right)$, where $x_y = (x, y)$ for clarity

From a solution found by the best hierarchical problem succession, we extract the corresponding genome architecture. Let $m, n \in \mathbb {N}{}$ be the number of repeats (pair of regions) and the number of single-copies, respectively. A genome contains $2\,m + n$ regions. Two items are sufficient to describe a genome with its regions:

$$\begin{aligned} COR = \left( {r_0, r_1, \dots , r_{m+n-1}}\right) \end{aligned}$$

contains the forward regions, i.e. it is a ($m+n$)-uplet of $m+n$ oriented contig sequences.

$$\begin{aligned} SOR&= \begin{pmatrix} rid_0&{} ror_0\\ \vdots &{} \vdots \\ rid_{2m+n-1} &{} ror_{2m+n-1} \end{pmatrix}\\&\in \left( {\mathbb {N}{} \times \{f, r\}{}}\right) ^{2m+n} \end{aligned}$$

is a linearised circular sequence of oriented regions. For each $i \in \llbracket {0,2\,m+n}\llbracket$, if $ror_i = f$ then $SOR[i]$ represents the forward region $COR[rid_i]$, else if $ror_i = r$ then $SOR[i]$ represents the reverse $\overline{COR[rid_i]}$. Figure 9 illustrates the regions extracted by Algorithm 4 in the toy example.

The key idea of such an extraction algorithm is to start from the starting vertex $s$ and walk over the chosen edges. During the walk, for each vertex we need to identify the type of its region, and then add the vertex to the corresponding region (to the corresponding region identifier $rid_r$). Algorithm 1 aims to determine the region type for a given vertex. The sequence of oriented regions $SOR$ begins by the starter’s region that is necessarily a single-copy (because $mult(contig(s)) = 1$). The first oriented contig may not be the starter. Algorithm 2 gives the *initial vertex* associated to the first oriented contig of the starter’s region. During the walk in the solution circuit from the initial vertex, a new region begins each time the region type changes. When the current vertex participates in a repeat, we must check if the next one participates in the same repeat, although the region type may not change (Algorithm 3). At the end, Algorithm 4 builds $COR$ and $SOR$ from an ILP solution.

To build the repeated regions, we use a First In First Out (FIFO) queue for the DRs, and a Last In First Out (LIFO) queue for the IRs. Each queue $rep\_queue$, is associated with three methods:

$$rep\_queue.{\textsc {put}}(x)$$
append $x$ to the FIFO/LIFO;
$$rep\_queue.{\textsc {is\_empty}}$$
returns true if the queue is empty;
$$rep\_queue.{\textsc {peek}}$$
returns the first/last value in the FIFO/LIFO;
$$rep\_queue.{\textsc {pop}}$$
deletes the first/last value in the FIFO/LIFO and returns it.

In the following, the given time complexities are under the assumption that the belonging test “*is *$x \in X$?” for an object $x$ in a set $X$ is in $\Theta {(1)}$. Algorithm 1 is in $\Theta {(1)}$. Algorithm 2 is in $O(|{SC_s}|)$, where $|{SC_s}|$ is the number of contigs composing the single-copy region that contains the starting vertex. Algorithm 3 is in $\Theta (1)$, and so Algorithm 4 is in $O(|{V}| + |{E}|)$.

**Algorithm 1:**
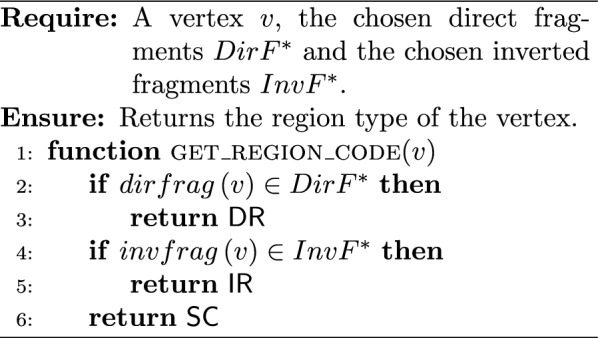
Get the region type for a given vertex

**Algorithm 2:**
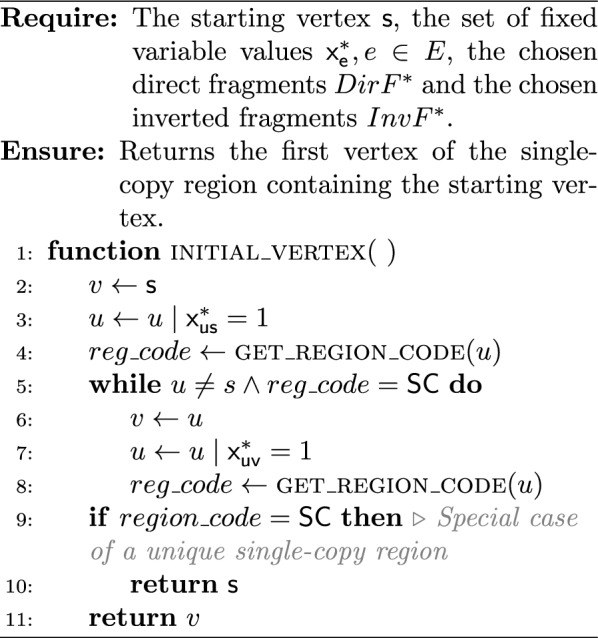
Get the initial vertex of the single-copy containing the starting vertex

**Algorithm 3:**
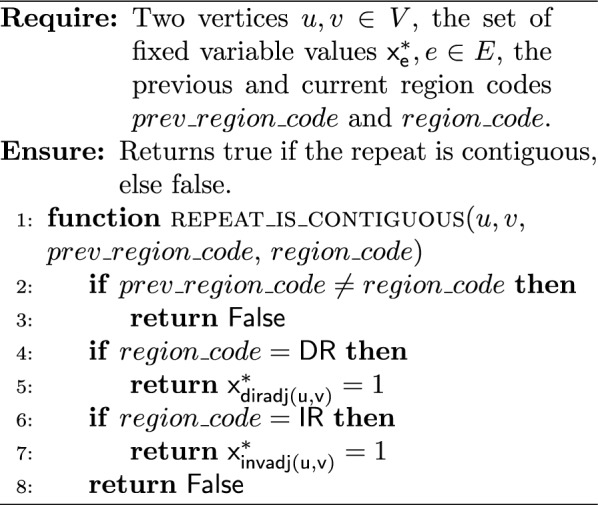
Is the repeat contiguous?

**Algorithm 4:**
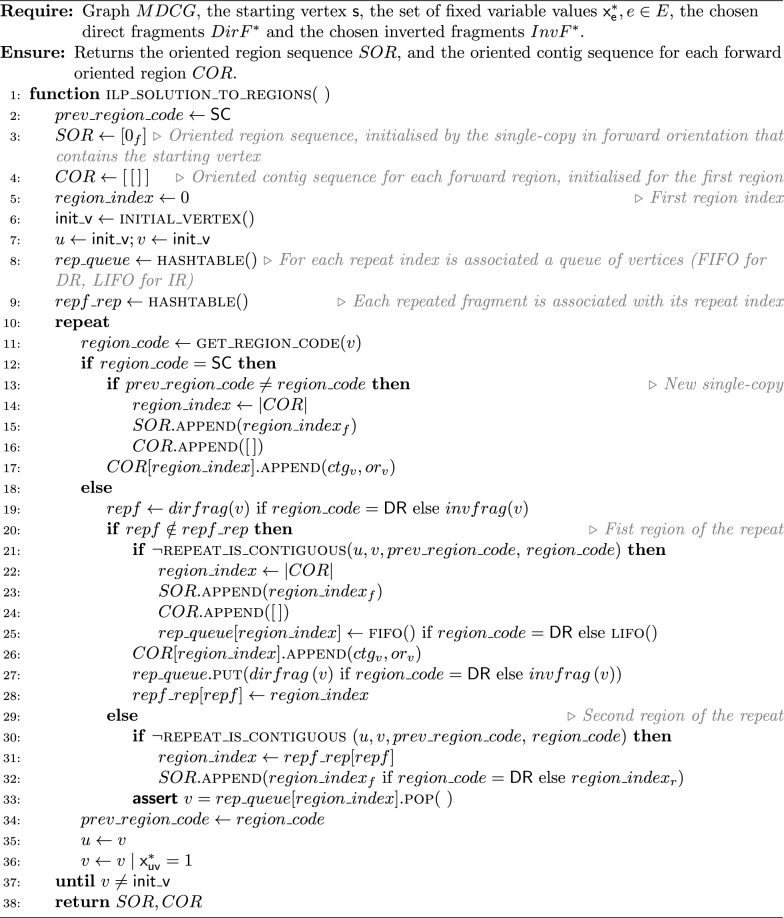
ILP solution to regions

## Multiple genome forms

The sequence of oriented regions $SOR$ represents one chloroplast genome form. Recall that especially with the LSC-IR-SSC-IR architecture (Fig. 1a), the SSC can be reversed during the DNA replication phase. In the following, our goal is to retrieve other forms from the one obtained by the hierarchical problem succession.

**Fig. 10:**
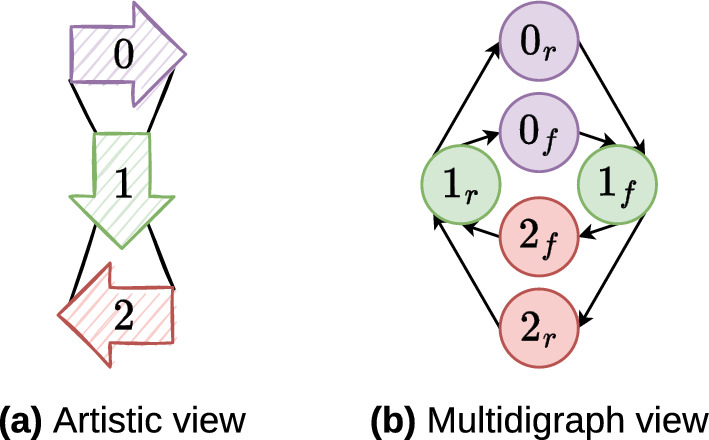
The toy example’s solution given in Fig. 9 results in the graph visualised in Fig. 5 will give a region graph that shows an LSC-IR-SSC-IR architecture as the one given in Fig. 1a. Two oriented region sequences (genome forms) are obtained by finding the eulerian circuits: $0_f \rightarrow 1_f \rightarrow 2_f \rightarrow 1_r$ and $0_f \rightarrow 1_f \rightarrow 2_r \rightarrow 1_r$. They correspond to two structural haplotypes commonly found in the chloroplast cells, and described in Fig. 2. **a** Each arrow represents a region. Each link connects two arrows’ extremity. Entering the tail/head and exiting the head/tail of an arrow corresponds to choosing the region in its forward/reverse orientation. **b** The same information is visualised. Each vertex is a region with a fixed orientation, and each edge connects two oriented regions

Towards this goal, we introduce a specific assembly graph: the *region graph*. The discovery of multiple genome forms is associated with the search of Eulerian circuits in this graph. Figure 10 illustrates the procedure for the toy example.

### Definition 26

(Region graph $RegGraph$) Given the $(m+n)$-uplet $COR$ of forward regions and the sequence $SOR$ of oriented regions, $RegGraph{} = (Vreg, Ereg, \Phi {})$ denotes a directed multigraph (multidigraph) named the region graph, such that:

$$\begin{aligned} Vreg = \left\{ \left. \begin{aligned}{}& v_{0,f}, \dots , v_{m+n-1,f}, \\&v_{0,r}, \dots , v_{m+n-1,r} \end{aligned} \right| |{COR}| = m+n \right\} \end{aligned}$$

is the set of oriented regions ($|{Vreg}| =2|{COR}|$). For each vertex, bijective function $vreg\colon Vreg \hookrightarrow{\!\!\!\!\!\!\!\!}\rightarrow \{ r \in COR\} \times \{ f,\,r\}$ $\{ r \in COR\} \times \{ f,\,r\}$ provides the oriented region it represents.

$Ereg$ is the multiset of links between two oriented regions in $SOR$ (including between the last and the first regions). Note that $\forall e \in Ereg$, $\overline{e} \in Ereg$ denotes its reverse where $\Phi {}(e) = \left( {u, v}\right)$, $\Phi {}(\overline{e}) = \left( {\overline{v}, \overline{u}}\right)$ ($|{Ereg}| = 2|{SOR}|$).

$\Phi {} \colon Ereg \rightarrow \{\left( {u, v}\right) \mid u, v \in Vreg\}$ is the incident function, such that for two consecutive oriented regions $r_i$ and $r_j$ in $SOR$ (including the last and the first ones), $\exists ! \, e \in Ereg \mid \Phi {}(e) = (vreg^{-1}(r_i),$ $vreg^{-1}(r_j))$.

Figure 10 illustrates the region graph for the toy example’s solution given in Fig. 9. Based on the above graph, we can find different sequences of oriented regions. Each region, independently of its orientation, in each sequence, must participate the same number of times.

### Definition 27

(Eulerian circuit in $RegGraph$) A circuit in $RegGraph{} = (Vreg, Ereg, \Phi {})$ is defined as Eulerian when:

- It begins from and ends with vertex $0_f$ representing the region containing the starter in forward orientation, i.e. $vreg(0_f) = COR[0]$;
- It passes through exactly one of the two versions of each edge ($e \in Ereg$ otherwise $\overline{e} \in Ereg$).

### Proposition 1

(An eulerian circuit is a valid oriented contig sequence for $RegGraph$) An Eulerian circuit in $RegGraph{} = (Vreg, Ereg, \Phi {})$ provides a valid sequence of oriented contigs (Definition 1).

### Proof

Let $RegGraph{} = (Vreg, Ereg, \Phi {})$ be a region graph. Denote by $euc = (v_0, v_1, \dots , v_{2\,m+n-1})$ an Eulerian circuit in $RegGraph$. For each two consecutive oriented regions $v_i, v_j$ in $euc$, there exists an edge $e \in Ereg$ such that $\Phi {}(e) = (v_i, v_j)$. According to Definition 26, $vreg(v_i) = r_i$ and $vreg(v_j) = r_j$ are also consecutive in the oriented region sequence that has originally built $RegGraph$, otherwise in its reverse. Thus, according to Algorithm 4, there is an edge $(u, v) \in E$ in $MDCG$ such that $(ctg_u, or_u)$ and $(ctg_v, or_v)$ equal $r_i[|{r_i}| - 1]$ and $r_j[0]$, respectively. $\square$

We can easily verify that the number of Eulerian circuits is bounded by $O(2^{m'})$, where $m' \le m$ is the number of inverted repeats.

Now, given a region graph $RegGraph$, finding all the Eulerian circuits is equivalent to retrieve all the possible chloroplast genome forms. Each Eulerian circuit traverses exactly the same regions, but not necessary in the same orientations. Figure 10 gives the resulting region graph obtained from the input data given in Table 1.

We accept all the Eulerian circuits, although they may contradict the repeated region interval constraints given in Definitions 10 and 11. For example, the oriented region sequence $(0_f, 1_f, 2_f, 3_f, 1_f, 3_r)$ respects the definitions, where $(1_f, 1_f)$ is a DR and $(3_f, 3_r)$ is an IR. One of the Eulerian circuit produces the oriented region sequence $(0_f, 1_f, 2_f, 3_f, 1_r, 3_r)$. Region $1_f$ now evolves in a new IR $(1_f, 1_r)$. The order between the regions of the two IRs contradicts Definition 11.

## $\mathcal{N}\mathcal{P}$-completeness

To prove the $\mathcal{N}\mathcal{P}$-completeness of one of the two hierarchical problem successions (decision version), it is sufficient to focus on only one scaffolding problem for the repeat. Here, we will focus on the decision version of $\mathcal {IRP}$ ($\mathcal {DIRP}$).

### Definition 28

($\mathcal {IRP}$ decision problem–$\mathcal {DIRP}$) Given a set of contigs $\mathcal {C}$, their multiplicities, a set of links $\mathcal {L}$, a starting contig $s$, two integers $k, m' \in \mathbb {N}{}$, is there a valid sequence of oriented regions for $\mathcal {IRP}$ with $\sum _{ir \in IR} replen(ir) \ge k$ and $|{IR}| \le m'$, where $IR$ is the solution set of inverted repeats?

### Proposition 2

($\mathcal {DIRP}$ is in $\mathcal{N}\mathcal{P}$) Given the input of $\mathcal {DIRP}$, a sequence of oriented regions $SOR$, the sequence of oriented contigs for each region $COR$, two integers $k, m' \in \mathbb {N}{}$. There is a polynomial time algorithm that checks if the given solution is valid and if its accumulative repeat length equals at least $k$ and the number of repeats equals at most $m'$.

### Proof

Algorithm 5 verifies if the sequence of oriented regions is valid. It requires two traversals of $SOR$: (i) to identify which regions in the sequence form IRs; (ii) to verify if the order between the IRs is valid (thanks to the use of a LIFO). It also checks if the associated accumulative repeat length equals at least $k$ and the number of repeats equals at most $m'$.

A LIFO $ir\_lifo$ is associated with four methods:

$$ir\_lifo.{\textsc {put}}(x)$$
append $x$ to the LIFO;
$$ir\_lifo.{\textsc {is\_empty}}$$
returns true if the LIFO is empty;
$$ir\_lifo.{\textsc {peek}}$$
returns the last value in the LIFO;
$$ir\_lifo.{\textsc {pop}}$$
deletes the last value in the LIFO and returns it.

Algorithm 6 verifies if the corresponding sequence of oriented contigs is valid. It first retrieves the total sequence of oriented contig $SOC$ from the sequence of oriented regions $SOR$ and the sequence of each forward regions $COR$. It traverses the oriented contigs in $SOC$, verify if each two consecutive oriented contigs are linked in the link set, and count the number of times a contig occur. At the end, it checks if each contig does not appear more than its multiplicity number of times.

It is straightforward to see that Algorithm 5 and Algorithm 6 are linear according the size of $SOR$ and $COR$. Remember we assume that the belonging test “*is *$x \in X$?” for an object $x$ in a set $X$ is in $\Theta {(1)}$. $\square$

**Algorithm 5:**
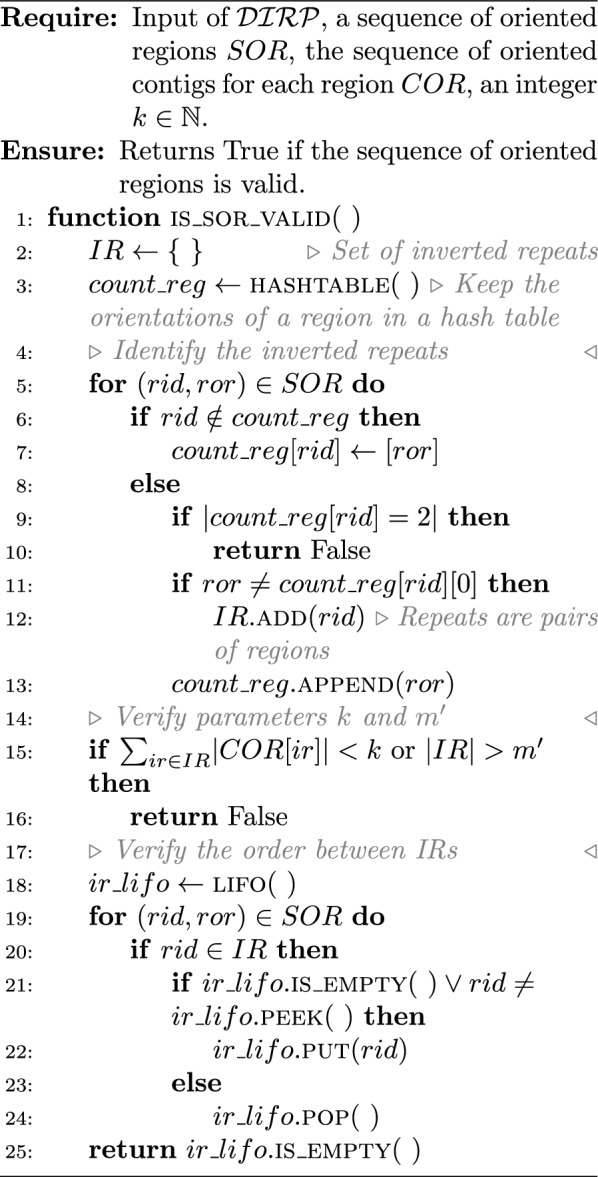
Verify the validity of the sequence of oriented regions

**Algorithm 6:**
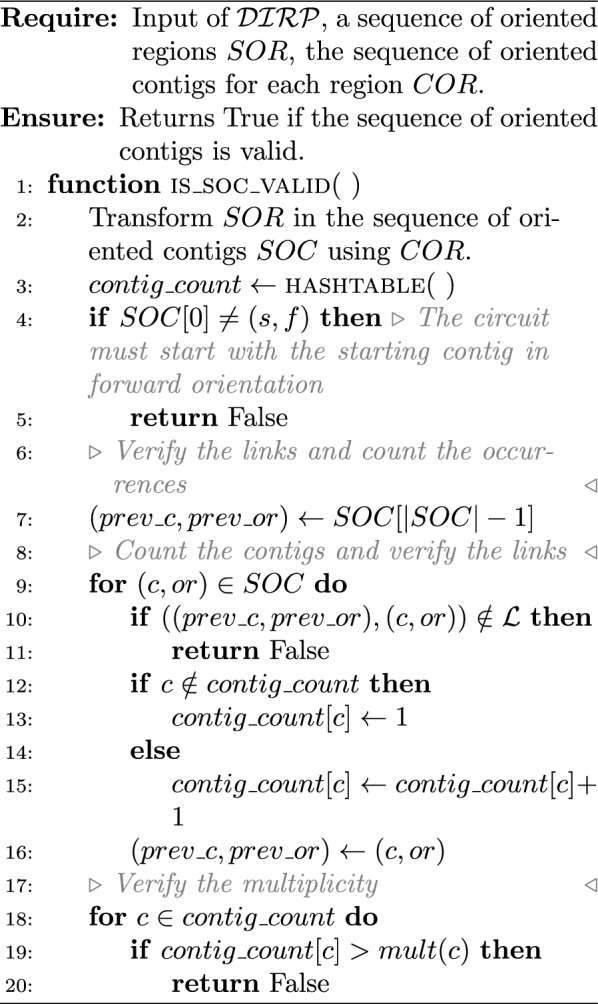
Verify the validity of the sequence of oriented contigs

### Proposition 3

$$\mathcal {DIRP}$$ is $$\mathcal{N}\mathcal{P}$$-Hard.

**Fig. 11:**
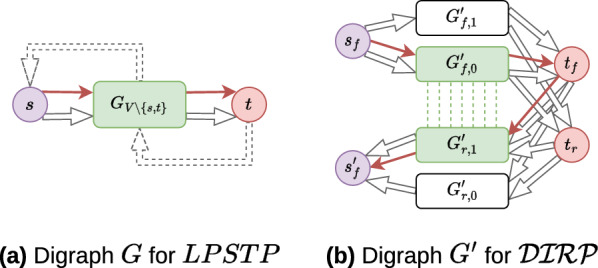
From a digraph $G$ for $LPSTP$ to a digraph $G'$ for $\mathcal {IRP}$. Bold red edges in both sub-figures correspond to the solution path for $LPSTP$ and $\mathcal {IRP}$ problems, respectively. **a** $G_{V {\setminus } \{s,t\}}$ is the subgraph induced by the vertex set $V {\setminus } \{s,t\}$. As the longest path exits $s$ and enters $t$, dashed edges do not participate in the solution. **b** Green dashed line between vertices in $G'_{f0}$ and $G'_{r1}$ visualise the inverted fragments

### Proof

By reduction from the longest path decision problem from vertex $s$ to vertex $t$ ($\mathcal {LPSTP}$), known to be $\mathcal{N}\mathcal{P}$-Complete [19].

Consider an instance $\mathbb {I} \in \mathcal {LPSTP}{}$ composed of a directed graph $G = \left( {V, E}\right)$, two vertices $s, t \in V$ and an integer $k \in \mathbb {N}{}$ (the hypothetical number of vertices between $s$ and $t$ in the longest path). We shall build an instance transform function $tf$ such that $\mathbb {I} \in \mathcal {LPSTP}{} \iff tf(\mathbb {I}) \in \mathcal {DIRP}{}$. Function $tf$ transforms graph $G$ to graph $G'= \left( {V', E'}\right)$, vertex $s \in V$ to vertices $s_f, s'_f \in V'$, $t \in V$ to $t_f, t_r \in V'$, $k$ to $k' = 2k$, and fix parameter $m' = 1$. Figure 11 illustrates the transformation.

All four subgraphs $G'_{or,i}$ in Fig. 11b can be seen as copies of $G_{V {\setminus } \left\{ s, t\right\} } = \left( {V {\setminus } \left\{ s, t\right\} , E_{V {\setminus } \left\{ s, t\right\} }}\right)$ (the subgraph induced by the vertex set $V {\setminus } \{s,t\}$) in Fig. 11a. For each $or \in \{f, r\}{}$, for each $i \in \{0, 1\}$, $G'_{or,i} = \left( {V'_{or,i}, E'_{or,i}}\right)$ such that:

- There is a bijective function $vtrans_{{or,i}} \colon V\backslash \{s,t\} \hookrightarrow{\!\!\!\!\!\!\!\!}\rightarrow V_{{or,i}}$ , where $\forall v \in V'_{or,i}$, $vor(v) = or$ and $vocc(v) = i$;
- There is a bijective function $etrans_{{or,i}} \colon E_{{V\backslash \{ s,t\} }} \hookrightarrow{\!\!\!\!\!\!\!\!}\rightarrow E^{\prime}_{{or,i}}$ where $\forall (u, v) \in E_{V {\setminus } \left\{ s, t\right\} }, \exists ! \, (u', v') \in E'_{or,i}$ such that:
  $$\begin{aligned} vtrans_{or,i}(u), vtrans_{or,i}(v) = {\left\{ \begin{array}{ll} u', v'\\ \quad \text {if } or = f \\ v', u'\\ \quad \text {if } or = r \end{array}\right. } \end{aligned}$$

There is a bijective function $vsttrans_{{st}} \colon \{ s,t\} \hookrightarrow{\!\!\!\!\!\!\!\!}\rightarrow \{ (s_{f} ,s^{\prime}_{f} ),\,(t_{f} ,t_{r} )\}$. There is a function $esttrans\colon \{(s, w) \in E\} \cup \{(u, t) \in E\} \rightarrow E'$ such that:

- $$\forall (s, w) \in E$$:
  $$\begin{aligned}{}&esttrans(s, w) = \\&\quad \{(s_f, vtrans_{f,i}(w)) \in V'^2 \mid i \in \{0,1\}\}\\&\quad \cup \{(vtrans_{r,i}(w), s'_f) \in V'^2 \mid i \in \{0,1\}\} \end{aligned}$$
- $$\forall (u, t) \in E$$:
  $$\begin{aligned}{}&esttrans(u, t) = \\&\quad \bigcup _{i \in \{0, 1\}} \left\{ \begin{pmatrix} vtrans_{f,i}(u) &{} t_f\\ t_f&{} vtrans_{r,i}(u) \end{pmatrix} \in V'^2 \right\} \\&\quad \cup \left\{ \begin{pmatrix} vtrans_{f,i}(u) &{} t_r\\ t_r&{} vtrans_{r,i}(u) \end{pmatrix} \in V'^2 \right\} \end{aligned}$$

It is straightforward to see that there exists an algorithm in $O(|{V}| + |{E}|)$ that computes this transform function.

The sets $InvF{}$, $PInvF{}$ and $AInvF{}$ are built based on $G'_{or,i}$ graphs. Inverted fragments are visualised by green dashed vertical lines in Fig. 11b, where $InvF{} = \left\{ (i, j) \in V_{f,0} \times V_{r,1} \mid vtrans_{f,0}(i) = vtrans_{r,1}(j)\right\}$.

As the adjacent inverted fragments associate only vertices in $V_{f,0}$ with those in $V_{r,1}$, the path that maximises the number of contiguous inverted fragments exits $s_f$, goes through $G'_{f,0}$ to $t_f$ (or $t_r$, it does not matter), and passes through $G'_{r,1}$ to $s'_f$.

Since $G'_{f,0}$ is a copy of $G_{V {\setminus } \left\{ s, t\right\} }$, while $G'_{r,1}$ is its reverse graph, there is a bijection between $V'_{f,0}$ and $V'_{r,1}$ vertices sets.

The way $G'$ is built implies only one IR is assembled (so parameter $m' = 1$ is respected). The length of this IR ($k' = 2k$) gives the hypothetical length of the longest path in $\mathcal {LPSTP}$.

To conclude, as there is a linear time complexity transform function $tf$ such that $\mathbb {I} \in \mathcal {LPSTP}{} \iff tf(\mathbb {I}) \in \mathcal {DIRP}{}$, $\mathcal {DIRP}$ is $\mathcal{N}\mathcal{P}$-Hard. $\square$

From Propositions 2 and 3 we conclude that $\mathcal {DIRP}$ is $\mathcal{N}\mathcal{P}$-Complete.

## Numerical results

We develop khloraascaf,^2^ a python package that computes the scaffolding of chloroplast contigs. It can either use Gurobi solver or CBC. All the following runs have been executed on a Linux laptop computer (32GB RAM, Intel^®^ Core™ i7–10610U CPU @ 1.80GHz $\times 8$). Each time, we select Gurobi solver.

### Complexity validation on artificial data

khloraascaf is also accessible as an API, that permits in this section to study the combinatorial behaviour of $\mathcal {IRP}$.

We demonstrate in Sect "[Sec Sec19]" that $\mathcal {DIRP}$ is in the general case $\mathcal{N}\mathcal{P}$-Complete. Furthermore, the heteroplasmy for the chloroplast genome is very often caused by the presence of an inverted repeat, that reverses the region(s) between it.

Thus, we artificially build a contig set with the associated attributes, and a link set, such that the genome architecture behind corresponds to the following circular sequence of oriented regions: $SC1-IR-SC2-\overline{IR}$. In the following, we run what corresponds to $\mathcal {IRP}$ computation in khloraascaf on two types of growing generated data: perfect and noisy artificial ones. To emphasise the effect of the inverted repeats in the complexity of $\mathcal {IRP}$, we fix the length of the single-copies, i.e. $|{SC1}| = |{SC2}| = |{SC}| = 20$, and we incrementally raise the length of the inverted repeat $|{IR}| = 20 k$ for $k \in \llbracket {1,10}\rrbracket$.

#### Perfect artificial data

The data generated for this section correspond to the smallest set of contigs, links, and the smallest multiplicities to make sure that $\mathcal {IRP}$ is feasible. The multiplied doubled contig graph associated with these perfect data has exactly the same topology as the one illustrated in Fig. 5. For instance, testing perfect artificial data acts as a control for further tests. Table 4 gives some Gurobi metrics.^3^

**Fig. 12:**
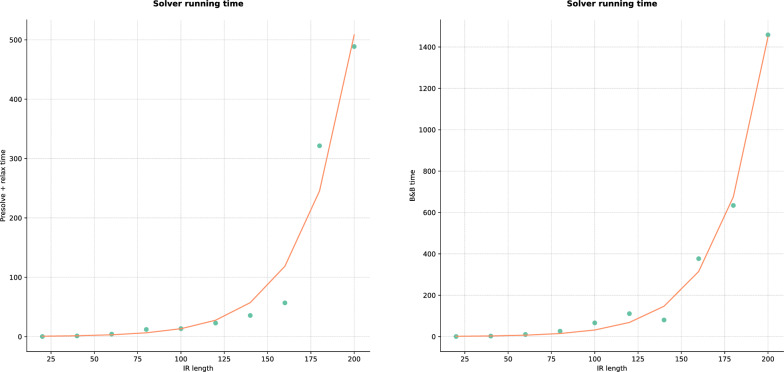
Solver running time distributions for perfect artificial data. Points are measured times, the red curves correspond to the best $ae^{bx}$ function applied on IR length axis

Observe that the gap is equal to 0 $\%$ and the problem is solved either during the presolving or the linear relaxation. Indeed, for the class of graph that contains only the perfect artificial data, there exist a polynomial algorithm. However, the relaxation time seems to fit an exponential distribution as well as for the B&B time, as shown in Fig. 12, even though the distributions should be treated with caution because of the limited number of points.

#### Noisy artificial data

Here we test the behaviour of the solver when we add noise to the perfect artificial data. In that case, for each generated contig, the multiplicity has 25 $\%$ chance to be overestimated by one (that increases $InvF$, $PInvF$ and possibly $AInvF$ sets). Similarly, for each contig, there is 25 $\%$ of chance to create a new link to another randomly chosen contig. This can generate more loops, and can increase the $AInvF$ set.^4^

As expected, now the gap is not ensured to be null, and some instances are not solved at the presolving or at the linear relaxation steps.

These numerical results corroborate the $\mathcal{N}\mathcal{P}$-Complete demonstration for a more general class of graphs (Table 5).

**Table 4:** Gurobi solver metrics on perfect artificial growing data

$\mathsf {|{}V|}$	$\mathsf {|{}E|}$	$\mathsf {|{}SC|}$	$\mathsf {|{}IR|}$	$|{\mathcal {L}}|$	Time	Opt.	% Gap	Nodes	Iter.
160	244	20	20	122	0.25	39.00	0.00	1	1242
					0.48	39.00			2944
240	404	20	40	162	1.23	79.00	0.00	1	4690
					2.54	79.00			10946
320	564	20	60	202	4.27	119.00	0.00	1	8945
					10.45	119.00			23864
400	724	20	80	242	12.15	159.00	0.00	1	15196
					26.29	159.00			39562
480	884	20	100	282	13.46	199.00	0.00	1	23109
					66.28	199.00			68740
560	1044	20	120	322	22.91	239.00	0.00	1	44048
					110.98	239.00			97646
640	1204	20	140	362	35.68	279.00	0.00	1	46071
					80.26	279.00			125084
720	1364	20	160	402	56.89	319.00	0.00	1	75371
					376.74	319.00			256831
800	1524	20	180	442	321.52	359.00	0.00	1	71971
					634.01	359.00			196905
880	1684	20	200	482	488.74	399.00	0.00	1	88157
					1458.66	399.00			236406

$\mathsf {|{V}|}$, $\mathsf {|{E}|}$, $\mathsf {|{SC}|}$, $\mathsf {|{IR}|}$ and $|{\mathcal {L}}|$ respectively stand for the number of vertices, edges, contigs in each single-copy region, contigs in each region of the inverted repeat, and links; Time: the presolve time plus the relaxation time (above) and the B&B time (below); Opt.: the linear relaxation bound $UB$ (above) and the integer optimal value $Opt$ (below); % Gap: the MIP gap equals $100\times \frac{(UB-Opt)}{UB}$; Nodes: number of explored B&B nodes; Iter.: number of iterations for the LP relaxation (above) and for the B&B phase (below)

**Table 5:** Gurobi solver metrics on noisy artificial growing data

$\mathsf {|{}V|}$	$\mathsf {|{}E|}$	$\mathsf {|{}SC1|}$	$\mathsf {|{}SC2|}$	$\mathsf {|{}IR|}$	$|{\mathcal {L}}|$	Time	Opt.	% Gap	Nodes	Iter.
186	372	20	20	20	152	1.01	39.00	0.00	1	2862
		26	24	43	30	5.16	39.00			6888
280	688	20	20	40	212	6.89	79.00	0.00	1	6727
		28	23	89	50	12.26	79.00			19930
366	946	20	20	60	262	42.52	123.50	3.64	1	16821
		25	26	132	60	79.28	119.00			50246
452	1208	20	20	80	320	90.06	161.50	1.54	1	27822
		22	23	181	78	295.22	159.00			129174
556	1366	20	20	100	322	196.04	199.00	0.00	1	42292
		23	24	231	40	244.41	199.00			92009
662	1804	20	20	120	412	1007.59	242.50	1.44	1	84639
		29	26	276	90	1434.16	239.00			210798
736	1946	20	20	140	454	1108.09	283.00	1.41	1	228198
		24	26	318	92	3540.79	279.00			691619
822	2212	20	20	160	514	1118.76	323.00	1.24	1	86592
		26	27	358	112	2449.55	319.00			287146
902	2362	20	20	180	542	1591.18	363.00	1.10	1	91936
		26	27	398	100	2576.6	359.00			269958
996	2656	20	20	200	602	2294.85	404.00	1.24	1	116315
		26	26	446	120	3747.02	399.00			351501

The column descriptions are the same as the one in Table 4. Except that because of the noise on multiplicities, the sum of contig multiplicity for each region can change: this is the value below the number of contig in columns $\mathsf {|{SC1}|}$, $\mathsf {|{SC2}|}$ and $\mathsf {|{IR}|}$. Similarly, because of the noise on the number of links, the below value for $\mathcal {L}$ corresponds to the number of noisy links

### Synthetic chloroplast input data

In this section, we aim to validate experimentally the relevance of our scaffolding problem definition by running khloraascaf on synthetic data.

#### Input data generation

Here we briefly describe our protocol for input data generation.^5^ 200 chloroplast genomes (selected in CpGDB^6^) were downloaded from the NCBI^7^. For each of them, a set of reads was generated. The contigs were generated with Minia [6], a de Bruijn graph assembly approach. The links correspond to $k$-mer paths in the resulting compacted de Bruijn graph (cDBG) that connect two contigs. Finally, 31 instances were selected for which various difficulties have been detected, e.g. extra-links in the link set or combination of repeats.

The starter is the contig for which the matK gene, usually found in a single-copy region, maps on.

To obtain the multiplicity of a given contig $c$, we sum the length of the alignments of the reads mapping on $c$ (we denote by $MapR_c$ the set containing the reads that map on $c$). This sum is defined as the coverage $cov_c$ of the contig $c$ by the reads:

$$\begin{aligned} cov_c = \sum _{read \in MapR_c} |{align_c^{read}}| \end{aligned}$$

Where $align_c^{read}$ is the sequence representing the alignment of the read $read$ on the contig $c$. Its length equals the number of nucleotides of $read$ that match on $c$ (identity or substitution). Then the multiplicity $mult(c)$ of $c$ is obtained by normalising its coverage $cov_c$ by this of the starter $s$, $cov_s$: $mult(c) = \max \left( {1, \Big \lceil {cov_c / cov_s - 0.1}\Big \rceil }\right)$. As the multiplicity is an upper-bound of the usage of contig, we prefer to round up the normalisation only if the decimal part is greater than 0.1.

The existence-weight $wex(c)$ for a contig $c$ is computed by counting the number of nucleotides of $c$ that are covered by at least a gene of protein from a chloroplast near-species, normalised by the length of $c$.

#### The evaluation’s metrics

For each synthetic instance, we know the sequence of the oriented contigs and the sequence of the oriented regions we search for. In the sequel we test our scaffolding approach and evaluate the obtained region graph. For each instance, for each optimisation problem combination, we provide the following metrics:

- The total number of eulerian circuits in the region graph (genome forms);
- How many of them coincide with the sequence of oriented contigs we search for;
- How many of them coincide with the sequence of oriented regions we search for.

Evaluating the sequence of the oriented contigs is stringent. On the other hand, although a result can be evaluated as a false one, we can still retrieve the sequence of the chloroplast genome by applying an alternative sequence. As a consequence, for each instance we use Quast [10] to evaluate the sequences associated to each genome form. As the genome reference is known, Quast tries to find the minimum number of differences (relocation, inversion, indels) between the reference and the sequence we provide. Three metrics are chosen to evaluate the best genome form for each problem succession:

- The genome fraction of the reference;
- The number of misassemblies;
- The number of local misassemblies.

For more detailed descriptions of Quast metrics, you can refer to Appendix C.1.

It would be expected that Quast metrics illustrate wrong assembly for the instances for which the sequence of the contigs, and a fortiori this of the regions, are not retrieved. Analogously, the instance that truthfully retrieves the sequences would have good Quast metric. However, these assertions may be contradicted because of the contig and the link sequences generation.

In Sect. "Initial version", we provide the two metric sets when khloraascaf is applied to the original synthetic data, while in Sect. "Modified version" the metrics are reported for a subset of modified synthetic data.

#### Initial version

Table 6 provides all the metrics defined above for the 31 instances. The instances are solved very quickly (solver times $< 4.5$ sec., Table 9). khloraascaf successfully founds the sequence of the oriented contigs in 20 of them and retrieves the sequence of the oriented regions in 28 of them.^8^ Three categories of failures are identified.

##### Wrong starting contig and multiplicities estimations

This is the category for which our approach is dependent and sensible. In the presented version, we have used a given starting contig, and a wrong one can lead to reduce all the multiplicities. This is the case for Begonia_pulchrifolia and Lamprocapnos_spectabilis for which the starters are contigs that normally participate into an IR, that contradicts our assumption that the starter participates in an SC.

Independently of a right starting contig, the multiplicity computation is sensible from the noise on the contig coverage by the reads. Agathis_dammara and Pelargonium_nanum both suffer from only one contig under-estimated multiplicity.

##### $\mathcal {IRP}$ ’s objective: maximising the cumulative length of the minimum number of repeats

The sequence of oriented regions for the Cucumix_hystrix’s reference contains the following sub-sequences: $\left( {\dots , IR, IR', \dots , \overline{IR'}, SC, \overline{IR}}\right)$. As $\mathcal {IRP}$ also aims to minimise the number of repeats, it results in the merge of $IR - IR'$ and consequently does not retrieve $SC$, normally inserted between $\overline{IR'}$ and $\overline{IR}$.

##### Model’s robustness

While the sequences of oriented contigs for the repeats are retrieved, the ones of the single-copies suffer from extra-links combined with low, sometimes null, weights. On the one hand, in case of null weights, the circuits in Lathyrus_pubescens and Triosteum_pinnatifidum do not pass through contigs that must participate in the SCs. On the other hand, Podocarpus_totara possesses two objective-equivalent subpaths in an SC.

Surprisingly, our tool khloraascaf reversed some subparts of single-copies. This is due to extra-links, but they are specifically caused by the existence of very short IRs hidden in them (remember that the links correspond to paths between pairs of contigs in the cDBG). This behaviour is observed in Carpodetus_serratus, Jasminum_tortuosum and Lophocereus_schottii.

##### Nucleotide sequences misassemblies

To avoid confusion, we always use nucl. seq. to denote “nucleotide sequence” to contrast with sequences of contigs/regions. In the following we analyse the instances presenting an unexpected behaviour for the Quast metrics, regarding if the sequences are found. Appendix Table 8 permits verifying if the contigs that participate in the sequence have been correctly assembled. Except for Lathyrus_pubescens where one local misassembly is due to the missing contig in the sequence, all the (local) missassemblies in Commiphora_foliacea, Eucommia_ulmoides, Juniperus_scopulorum, Musa_ornata, Sciadopitys_verticillata, Taxus_baccata, Welwitschia_mirabilis provided by Quast are found in the nucl. seq. of links.

**Table 6:** Sequence and Quast metrics for the initial synthetic data version

			Successful		Quast		
Instance	ILPs	Total	$\textsf{SOC}$	$\textsf{SOR}$	%gnm	#mis	
Abies_alba	dr-sc	1	0	0	99.879	3	0
	ir-sc	2	1	2	100.0	0	0
Acorus_americanus	ir-sc	2	1	2	99.987	0	0
Agathis_dammara	dr-ir-sc	10	0	2	80.509	1	2
	ir-dr-sc	6	0	0	80.935	2	2
Azima_tetracantha	ir-sc	2	1	2	99.987	0	0
Begonia_pulchrifolia	–	–	–	–	–	–	–
Carpodetus_serratus	ir-sc	2	0	2	99.998	2	0
Circaeaster_agrestis	ir-sc	2	1	2	99.987	0	0
Clematis_repens	ir-sc	2	1	2	100.0	0	0
Commiphora_foliacea	ir-sc	4	1	4	98.82	0	5
Cucumis_hystrix	ir-sc	2	0	0	100.0	0	0
Eucommia_ulmoides	ir-sc	2	1	2	99.988	0	2
Jasminum_tortuosum	ir-sc	2	0	2	99.992	2	0
Juniperus_scopulorum	sc	1	1	1	99.722	0	1
Lamprocapnos_spectabilis	dr-sc	1	0	0	35.86	3	0
Lathyrus_pubescens	dr-sc	1	0	0	98.471	2	2
	ir-sc	2	0	2	98.496	0	2
Lophocereus_schottii	sc	1	0	1	99.884	1	0
Musa_ornata	ir-sc	2	1	2	99.97	0	2
Oenothera_glazioviana	ir-sc	2	1	2	100.0	0	0
Pelargonium_nanum	ir-sc	2	0	2	96.44	4	0
Podocarpus_totara	sc	1	0	1	99.712	4	0
Porphyra_purpura	dr-sc	1	1	1	100.0	0	0
Sagittaria_trifolia	ir-sc	2	1	2	100.0	0	0
Sciadopitys_verticillata	dr-sc	1	0	0	98.989	6	3
	ir-sc	2	1	2	98.997	1	4
Sciaphila_densiflora	sc	1	1	1	100.0	0	0
Selaginella_kraussiana	dr-sc	1	1	1	100.0	0	0
Selaginella_vardei	dr-sc	1	1	1	100.0	0	0
Taxus_baccata	sc	1	1	1	99.767	0	2
Triosteum_pinnatifidum	ir-dr-sc	2	0	2	99.278	0	2
Uvaria_macrophylla	ir-sc	2	1	2	99.896	0	0
Welwitschia_mirabilis	ir-sc	2	1	2	100.0	0	1
Wolffia_australiana	ir-sc	4	1	4	99.988	0	0

For each instance in the column Instance: ILPs provides the optimal (at most two) hierarchical problem successions; Total reports the number of eulerian circuits in the region graph (genome forms); $\textsf{SOC}$ is the number of oriented contig sequences that equal to the reference oriented contig sequence; $\textsf{SOR}$ is the number of oriented region sequences in bijection with the reference oriented region sequence; %gnm is the genome fraction of the best sequence produced by one of the genome forms; #mis are the number of misassemblies (left) and of local misassemblies (right)

#### Modified version

In this section, we present a manually changed synthetic data version to succeed the scaffolding. The goal is to precisely evaluate the robustness of khloraascaf. We detail the modifications bellow^9^:

- Agathis_dammara The multiplicity of contig $1$ is raised to be equal to 3.
- Begonia_pulchrifolia Contig $4$ becomes the starter. So according to the multiplicity computation described in Sect. "Input data generation", the multiplicities of contigs $1$ to $5$ become 1, while this one of contig $0$ raises to 2.
- Carpodetus_serratus Link $(10_r, 11_f)$ is deleted.
- Jasminum_tortuosum Link $(6_f, 4_f)$ is added.
- Lamprocapnos_spectabilis Contig $8$ becomes the starter. So the multiplicities of contigs $2$, $4$, $6$, $10$ and $11$ increase by one, while the ones of contigs $0$, $1$ and $3$ increase by two.
- Lathyrus_pubescens The weight of contig $12$ raises to $0.01$.
- Lophocereus_schottii Link $(10_f, 3_f)$ is deleted.
- Pelargonium_nanum The multiplicity of contig $2$ raises from 3 to 4, without respecting the computation of the multiplicity described in Sect. "Input data generation".
- Podocarpus_totara Link $(6_f, 11_r)$ is deleted.
- Triosteum_pinnatifidum The weight of contig $7$ raises to $0.01$.

Table 7 provides all the metrics obtained by running khloraascaf. The instances are also solved very quickly (solver times $< 3$ sec., Table 10). It truthfully finds all the sequences for the modified synthetic data except for Agathis_dammara instance. Although all the repeats (both the direct and the inverted ones) have been retrieved, one of the single-copy region has not been found. It can be explained by extra-links that create alternative paths with the same optimal value for $\mathcal {SCP}$. Note that the oriented region sequence has been still retrieved.

All the (local) misassemblies provided by Quast for Carpodetus_serratus, Lathyrus_pubescens, Pelargonium_nanum and Triosteum_pinnatifidum just concern the nucl. seq. of links that is not a khloraascaf issue.^10^

**Table 7:** Sequence and Quast metrics for the modified synthetic data version

			Successful		Quast		
Instance	ILPs	Total	$\textsf{SOC}$	$\textsf{SOR}$	%gnm	#mis	
Agathis_dammara	dr-ir-sc	10	0	2	99.973	3	2
	ir-dr-sc	6	0	0	99.986	2	2
Begonia_pulchrifolia	ir-sc	2	1	2	99.988	0	0
Carpodetus_serratus	ir-sc	2	1	2	100.0	0	1
Jasminum_tortuosum	ir-sc	2	1	2	99.992	0	0
Lamprocapnos_spectabilis	ir-sc	2	1	2	99.633	0	3
Lathyrus_pubescens	dr-sc	1	0	0	99.007	2	4
	ir-sc	2	1	2	99.731	0	1
Lophocereus_schottii	sc	1	1	1	99.997	0	0
Pelargonium_nanum	ir-sc	2	1	2	97.661	1	2
Podocarpus_totara	sc	1	1	1	99.754	0	0
Triosteum_pinnatifidum	ir-dr-sc	2	1	2	99.525	0	2

The caption is the same as in Table 6

## Conclusion

While the scaffolding problem is traditionally defined with distances data between the contigs, we show it is possible to avoid them in the case of the well-studied circular chloroplast genomes. Based on their specificities, we provide a new scaffolding formulation focused on revealing structural haplotypes.

Under the assumption that chloroplast genomes possess few repeats, we formalise their architectures as combinations of direct and inverted repeats, joined by single-copies, where the repeats are couples of identical (or reversed) nucleotide sequences. We tackle the chloroplast genome scaffolding as a discrete optimisation problem that yields three suboptimisation ones. We split the inherent multi-objective problem into one optimisation problem per region type. As a consequence, it is necessary to choose the order of subproblem resolutions as a function of the results of previously solved problems. This is what has been addressed through the hierarchical combination strategy. We model each subproblem with an ILP.

As our dedicated chloroplast scaffolding definition is a region-scaffolding-driven, the region graph is a natural data structure to reveal distinct genome forms that can coexist in a same cell. Indeed, particularly due to an IR flip-flop mechanism, regions between the IRs can be reversed during the genome replication process.

Moreover, we prove the decision version of the Chloroplast Scaffolding Problem ($\mathcal {CHSP}$) to be $\mathcal{N}\mathcal{P}$-Complete in the general case, even though numerical results on perfect artificial data suggest there is a class of $MDCG$ graphs where the problem is in $\mathcal {P}$. Without surprise, the noisy artificial data benchmark confirms the theoretical complexity.

We have implemented our approach and the ILP formulations in a Python3 package, khloraascaf,^11^ that we test on synthetic chloroplast contigs and links data. When the input data permit finding the solution, khloraascaf successfully retrieves the genome forms. Even if the decision problem is $\mathcal{N}\mathcal{P}$-Complete, the small size of the input data enables to quickly solve optimally $\mathcal {CHSP}$.

Our results show that the scaffolding-repeat problem formulations $\mathcal {DRP}$ and $\mathcal {IRP}$ seem to be sufficient to scaffold the repeats. This tends to validate our assumptions on the small number of repeats, and especially on the sufficiency of defining the repeats as pairs of equal (reversed) nucleotide sequences.

## Discussion and perspectives

While our scaffolding problem formulation seems to be sufficient to retrieve the repeats, it seems it is not fully suitable for single-copies. If we have applied the maximum-weighted circuit problem to scaffold the single-copies after having scaffolded the repeats, it was only with the intention of staying in the context of global optimisation. On the one hand, having weights on links may have been more appropriate than just considering weights on the contigs: in some sense, that is the purpose of distances. On the other hand, khloraascaf could initially scaffold the repeats and then propose several solutions to link them, e.g. scored by the weights.

Concerning the tests on synthetic data, we should use a more traditional assembly graph input: in fact, as revealed by comparing the khloraascaf results with the reference genomes, the used link generation suffers from local, or worse, global, misassemblies. As next step, we plan to inject khloraascaf into a state-of-the-art chloroplast genome pipeline, like GetOrganelle, and substitute what can be identified as the scaffolding part by our method. Hence, we should be able to relevantly compare khloraascaf approach with the state-of-the-art.

khloraascaf is sensible to the contig multiplicity computation. For now, a contig multiplicity is obtained by normalising its coverage by this of the starter. A better strategy may be to choose the smallest coverage for the normalisation as we expect the multiplicities to be upper-bounds of the contigs use.

Another issue concerns the choice of the starter: while it depends on the result of the mapping of matK gene map on the contigs, $\mathcal {DRP}$, $\mathcal {IRP}$ and $\mathcal {SCP}$ problems may be adapted for a set of candidate for the starter.

To generalise $\mathcal {CHSP}$ on non-equally (reversed) pair of regions for the repeats, two combining ideas are proposed: on the one hand, we can add pairs of contigs to the repeated fragment sets from the user-input. On the other hand, $\mathcal {CHSP}$ should be able to handle the case when a single-copy region is in only one of the regions of a repeat: for now, the contiguity constraint and the objective exclude this case. The contiguity constraint can be adapted to accept only one contiguous region out of the two.

From a user-case perspective, the region graph data structure can be used to determine what genome forms are present in the read set, and in which proportion. Indeed, as the region graph explicitly describes the junctions between the regions, especially between the inverted repeats, one may extract the nucleotide sequences of these junctions to answer the existence of the forms in the read set.

## Data Availability

The Python3 package khloraascaf can be installed with PyPI at https://pypi.org/project/khloraascaf/ and the numerical results can be reproduced thanks to the instructions given in https://khloraascaf-results.readthedocs.io/en/latest/benchmark_3/.

## Appendix A

**Repeated fragment set functions**

$$\begin{aligned} dirfrag \colon &\left\{ v \in p, \forall p \in DirF{}\right\} \rightarrow DirF{} \\&v \mapsto {\left\{ \begin{array}{ll} (v, v') \quad \text {if } occ_v \mid 2 \\ \text {where } occ_{v'} = occ_v + 1 \\ (v', v) \quad \text {else } occ_v \not \mid 2 \\ \text {where } occ_{v'} = occ_v - 1 \\ or_v = or_{v'} \end{array}\right. } \\ invfrag \colon &\left\{ v \in p, \forall p \in InvF{}\right\} \rightarrow InvF{} \\&v \mapsto {\left\{ \begin{array}{ll} (v, v') \quad \text {if } or_v = 0\\ \text {where } or_{v'} = 1 \\ \quad \wedge occ_{v'} = occ_v + 1 \\ (v', v) \quad \text {else } or_v = 1 \\ \text {where } or_{v'} = 0 \\ \quad \wedge occ_{v'} = occ_v - 1 \end{array}\right. } \\ diradj\colon E&\rightarrow E \\ (u, v)&\mapsto (u', v') \\&\quad ctg_{u'} = ctg_u\\&\quad or_{u'} = or_u \\&\quad occ_{u'} = {\left\{ \begin{array}{ll} occ_u + 1 \text { if } occ_u \mid 2 \\ occ_u - 1 \text { otherwise} \end{array}\right. } \\&\quad ctg_{v'} = ctg_v\\&\quad or_{v'} = or_v \\&\quad occ_{v'} = {\left\{ \begin{array}{ll} occ_v + 1 \text { if } occ_v \mid 2 \\ occ_v - 1 \text { otherwise} \end{array}\right. } \\ invadj \colon E&\rightarrow E \\ (u, v)&\mapsto (v', u') \\&\quad ctg_{v'} = ctg_v\\&\quad or_{v'} = 1 - or_v\\&\quad occ_{v'} = 2 \left\lfloor \frac{occ_v}{2}\right\rfloor + (1 - or_v) \\&\quad ctg_{u'} = ctg_u\\&\quad or_{u'} = 1 - or_u\\&\quad occ_{u'} = 2 \left\lfloor \frac{occ_u}{2}\right\rfloor + (1 - or_u) \end{aligned}$$

## Appendix B

**Reduction of the repeated fragment sets**

In this section, we give prove the minimality and the sufficiency of the repeated fragment sets ($RepF{}, PRepF{}, ARepF{}$) definitions.

### B.1 Repeated fragment set reductions

Here we consider the reduction operations for $DirF$ and $InvF$, and conclude on their minimality. Only two vertices $i, j \in V$ with the same identifier, i.e. $ctg_i = ctg_j$ can form a repeated fragment. The relative orientations between $i$ and $j$ is determined according to the type of the repeat the fragment is associated to. The occurrences of the two vertices in a repeated fragment must differ.

Two combinatorial reductions are necessary—*commutative* and *pairing* reductions:

i. commutative reduction means that $\forall (i, j) \in RepF{}, (j, i) \notin RepF{}$;
ii. pairing reduction means that it is not necessary to pair all the occurrences cross all the occurrences, and we can group by consecutive occurrences without any intersection.

Reminder of the $DirF$ definition:

$$\begin{aligned} DirF{} = \bigcup _{c \in \mathcal {R}{}} \left\{ \begin{aligned}{}&(i, j) \in V^2 \text { s.t.} \\&\quad ctg_i = ctg_j = c \\&\quad \wedge or_i = or_j \in \{f, r\}{} \\&\quad \wedge occ_i = occ_j - 1 = 2 k\\&\quad 0 \le k < \left\lfloor {\frac{mult(c)}{2}}\right\rfloor \end{aligned}\right\} \end{aligned}$$

#### Proposition 4

(Commutative reduction for $DirF$)

$$\begin{aligned} \forall (i, j) \in DirF{}, (j, i) \notin DirF{} \end{aligned}$$

#### Proof

Let $$(i, j) \in DirF{}$$. $$occ_j = occ_i + 1$$ so $$(j, i) \notin DirF{}$$ $$\square$$

#### Proposition 5

The direct fragment set contains a sufficient number of direct fragments to form all the solutions.

#### Proof

The idea is to find the minimum occurrence difference between two direct fragments such that they can both be chosen to form DRs. Let $(i, j) \in DirF{}$ be a direct fragment and suppose that it is chosen to participate into a DR. Let $k, l \in V \mid ctg_k = ctg_l = ctg_i = ctg_j \wedge or_k = or_l = or_i = or_j$ be a candidate for the next direct fragment:

- the occurrences of $k$ and $l$ must differ from the ones of $i$ and $j$, as a vertex can occupy at most one position, so $occ_j < occ_k$;
- according to Proposition 4, $occ_k < occ_l$ and since occurrences are integers, $occ_k = occ_l - 1$.

So $occ_i = occ_j - 1 \le occ_k - 2 = occ_l - 3 \implies occ_i + 3 \le occ_l$. The smallest possible values for $occ_k$ and $occ_l$ equal $occ_i + 2$ and $occ_j + 2$. $\square$

#### Proposition 6

Direct fragment set $DirF$ is a minimal set for $\mathcal {DRP}$.

#### Proof

Firstly, we must keep the direct fragment for both the forward ($or_i = or_j = 0$) and the reverse orientations ($or_i = or_j = 1$) as the scaffolding problem aims to order and orientate the contigs, so by definition we cannot determine which orientation will participate in the order. Secondly, making step bigger than $2k$ prevents to use all the occurrences of a contig, so it contradicts the contig multiplicity definition. $\square$

Reminder of the $InvF$ definition:

$$\begin{aligned} InvF{} = \bigcup _{c \in \mathcal {R}{}} \left\{ \begin{aligned}{}&(i, j) \in V^2 \text { s.t.} \\&\quad ctg_i = ctg_j = c \\&\quad \wedge or_i = f \wedge or_j = r \\&\quad \wedge occ_i = occ_j - 1 = 2 k\\&\quad 0 \le k < \left\lfloor {\frac{mult(c)}{2}}\right\rfloor \end{aligned}\right\} \end{aligned}$$

#### Proposition 7

(Commutative reduction for $InvF$)

$$\begin{aligned} \forall (i, j) \in InvF{}, (j, i) \notin InvF{} \end{aligned}$$

#### Proof

Let $$(i, j) \in InvF{}$$. $$occ_j = occ_i + 1$$ so $$(j, i) \notin InvF{}$$ $$\square$$

#### Proposition 8

(Pairing reduction for $InvF$) The inverted fragment set contains a sufficient number of inverted fragments to form all the solutions.

#### Proof

The idea is to find the minimum occurrence difference between two inverted fragments such that they can both be chosen to form IRs. Let $(i, j) \in InvF{}$ be an inverted fragment and suppose that it is chosen to participate into an IR. Let $k, l \in V \mid ctg_k = ctg_l = ctg_i = ctg_j \wedge or_k \ne or_l$ be a candidate for the next direct fragment:

- to respect the property given by Proposition 7, $or_k = 1 - or_l = 0$
- the occurrences of $k$ and $l$ must differ from the ones of $i$ and $j$ when their orientations are matching, as a vertex can occupy at most one position. Furthermore, for a given occurrence and a given identifier, the two orientations are mutually exclusive, so $occ_j < occ_k$;
- according to Proposition 7, $occ_k < occ_l$ and since occurrences are integers, $occ_k = occ_l - 1$.

So $occ_i = occ_j - 1 \le occ_k - 2 = occ_l - 3 \implies occ_i + 3 \le occ_l$. The smallest possible values for $occ_k$ and $occ_l$ equal $occ_i + 2$ and $occ_j + 2$. $\square$

#### Proposition 9

Inverted fragment set $InvF$ is a minimal set for $\mathcal {IRP}$.

#### Proof

Step bigger than $2k$ prevents to use all the occurrences of a contig, so it contradicts the contig multiplicity definition. $\square$

### B.2 Pairs of repeated fragment set reductions

Here we consider the reduction operations for $PDirF$ and $PInvF$, and conclude on their minimality. Just the *commutative reduction* is necessary.

Reminder of pairs of direct fragments set definition:

$$\begin{aligned} PInvF{} = \left\{ \begin{aligned}{}&\big ((i, j), (k, l)\big ) \in InvF{}^2 \text { s.t.} \\&\quad ctg_j< ctg_k \\&\quad \vee \\&\quad ctg_j = ctg_k \wedge occ_j < occ_k \end{aligned}\right\} \end{aligned}$$

#### Proposition 10

(Commutative reduction for $PDirF$)

$$\begin{aligned} \forall ((i, j), (k, l)) \in PDirF{}, ((k, l), (i, j)) \notin PDirF{} \end{aligned}$$

#### Proof

Let $$((i, j), (k, l)) \in PDirF{}$$:

- if $ctg_j < ctg_k$ thus $ctg_l > ctg_i$ so $((k, l), (i, j)) \notin PDirF{}$
- else $ctg_j = ctg_k \wedge occ_j < occ_k$ thus $occ_l > occ_i$ so $((k, l), (i, j)) \notin PDirF{}$

$$\square$$

#### Proposition 11

Pair of direct fragment set $PDirF$ is a minimal set for $\mathcal {DRP}$.

#### Proof

By *reductio ad absurdum*: if $PDirF{}$ can be minimised to a $PDirF{}'$ set, then there exists a pair of direct fragments $(p, q) \in PDirF{} \mid (p, q) \notin PDirF{}'$.

Let $p = (i, j)$ and $q = (k, l)$, and let $q_2 = (m, n) \mid (p, q_2) \in PDirF{}' \wedge (q, q_2) \in PDirF{}'$. $i, j, k, l, m, n$ vertices can simultaneously participate in the solution.

Let build a counter-example: suppose that we have the order $i \, k \, l \, j \, m \, n$. This implies the direct fragments $p$ and $q$ fall into a forbidden case for $\mathcal {DRP}$, but this is not detected by Constraints C14 and C15 because $(p,q) \notin PDirF{}'$. According to Constraint C16, the direct fragments $p$ and $q_2$ can both match, and direct fragments $q$ and $q_2$ too. So $m_p = m_q = m_{q_2} = 1$: absurd because $p$ and $q$ are nested so $m_p$ and $m_q$ should be equal to $0$. $\square$

Reminder of pairs of inverted fragments set definition:

$$\begin{aligned} PInvF{} = \left\{ \begin{aligned}{}&\big ((i, j), (k, l)\big ) \in InvF{}^2 \text { s.t.} \\&\quad ctg_j< ctg_k \\&\quad \vee \\&\quad ctg_j = ctg_k \wedge occ_j < occ_k \end{aligned}\right\} \end{aligned}$$

#### Proposition 12

(Commutative reduction for $PInvF$)

$$\begin{aligned} \forall ((i, j), (k, l)) \in PInvF{}, ((k, l), (i, j)) \notin PInvF{} \end{aligned}$$

#### Proof

Let $$((i, j), (k, l)) \in PInvF{}$$:

- if $ctg_j < ctg_k$ thus $ctg_l > ctg_i$ so $((k, l), (i, j)) \notin PInvF{}$
- else $ctg_j = ctg_k \wedge occ_j < occ_k$ thus $occ_l > occ_i$ so $((k, l), (i, j)) \notin PInvF{}$

$$\square$$

#### Proposition 13

Pair of inverted fragment set $PInvF$ is a minimal set for $\mathcal {IRP}$.

#### Proof

By *reductio ad absurdum*: if $PInvF{}$ can be minimised to a $PInvF{}'$ set, then there exists a pair of inverted fragments $(p, q) \in PInvF{} \mid (p, q) \notin PInvF{}'$.

Let be $p = (i, j)$ and $q = (k, l)$, and let $q_2 = (m, n) \mid (p, q_2) \in PInvF{}' \wedge (q, q_2) \in PInvF{}'$. $i, j, k, l, m, n$ vertices can simultaneously participate in the solution.

Let build a counter-example: suppose that we have the order $i \, k \, j \, l \, m \, n$. This implies the inverted fragments $p$ and $q$ fall into a forbidden case for $\mathcal {DRP}$, but this is not detected by Constraints C14 and C15 because $(p,q) \notin PInvF{}'$. According to Constraint C16, the inverted fragments $p$ and $q_2$ can both match, and inverted fragments $q$ and $q_2$ too. So $m_p = m_q = m_{q_2} = 1$: absurd because $p$ and $q$ intersect so $m_p$ and $m_q$ should be equal to $0$. $\square$

### B.3 Adjacent repeated fragment set reductions

Here we consider the reduction operations for $ADirF$ and $AInvF$, and conclude on their minimality.

Reminder of adjacent direct fragments set definition:

$$\begin{aligned} ADirF{}= & {} {}&\left\{ \begin{aligned}{}&(u, v) \in E \text { s.t.} \\&\quad ctg_u \ne ctg_v \\&\quad \wedge occ_u = 2 k \\&\quad 0 \le k< \left\lfloor {\frac{mult_u}{2}}\right\rfloor \\&\quad \wedge occ_v = 2 k'\\&\quad 0 \le k'< \left\lfloor {\frac{mult_v}{2}}\right\rfloor \\ \end{aligned} \right\} \\&\bigcup{} & {} \left\{ \begin{aligned}{}&(u, v) \in E \text { s.t.} \\&\quad ctg_u = ctg_v \\&\quad \wedge (or_u = f \vee or_v = f)\\&\quad \wedge occ_u = 2 k\\&\quad \wedge occ_v = 2 k' \\&\quad 0 \le k< k' < \left\lfloor \frac{mult_u}{2}\right\rfloor \end{aligned} \right\} \end{aligned}$$

This set is the minimum exhaustive set of canonical edges which represent adjacent direct fragments. Two combinatorial reductions are necessary—*canonical reduction for different identifiers* (Propositions 14 and 15) and *same identifiers occurrences* reductions (Propositions 16 and 17).

#### Proposition 14

(Canonical reductions for $ADirF$ for different identifiers) $ADirF$ set contains only two of the eight edges that exist between two adjacent direct fragments when the vertex’ identifiers are different, i.e.

$$\forall (i, j) \in DirF{}, \forall (k, l) \in DirF{} \mid ctg_i \ne ctg_k$$:

$$\begin{aligned} \begin{array}{l} (i, k) \in E \\ \iff (i, l) \in E \\ \iff (j, k) \in E \\ \iff (j, l) \in E \end{array}&\iff \begin{array}{l} (i, k) \in ADirF{} \\ \wedge (\overline{k}, \overline{i}) \in ADirF{} \\ \wedge (i, l) \notin ADirF{}\\ \wedge (\overline{l}, \overline{i}) \notin ADirF{} \\ \wedge (j, k) \notin ADirF{}\\ \wedge (\overline{k}, \overline{j}) \notin ADirF{} \\ \wedge (j, l) \notin ADirF{}\\ \wedge (\overline{l}, \overline{j}) \notin ADirF{} \end{array} \\\\ \begin{array}{l} (k, i) \in E \\ \iff (k, j) \in E \\ \iff (l, i) \in E \\ \iff (l, j) \in E \end{array}&\iff \begin{array}{l} (k, i) \in ADirF{} \\ \wedge (\overline{i}, \overline{k}) \in ADirF{} \\ \wedge (k, j) \notin ADirF{}\\ \wedge (\overline{j}, \overline{k}) \notin ADirF{} \\ \wedge (l, i) \notin ADirF{}\\ \wedge (\overline{i}, \overline{l}) \notin ADirF{} \\ \wedge (l, j) \notin ADirF{}\\ \wedge (\overline{j}, \overline{l}) \notin ADirF{} \end{array} \end{aligned}$$

#### Proof

The reciprocal are trivial as $ADirF{} \subset E$.

All the edges using only $i$, $\overline{i}$, $k$ or $\overline{k}$ belong to $ADirF$ as all the vertex’ occurrences are even. While all the edges using $j$, $\overline{j}$, $l$ or $\overline{l}$ cannot belong to $ADirF$ as one of the vertex’ occurrence is odd. $\square$

#### Proposition 15

(Minimum $ADirF$ set for different identifiers) When the vertex’ identifiers are different, it is not possible to reduce the number of canonical edges based on occurrences comparisons.

#### Proof

In order to reduce $ADirF$ when the vertex’ identifiers are different, we have to be more restrictive on the occurrences constraints. To reduce the loops on $k$ and $k'$, we have to find an order between the occurrences of $u$ and $v$.

However, for each order relation between the occurrences ($occ_u \le occ_v$ and $occ_u \ge occ_v$), we can find two associated counter-examples. For the sake of clarity, we will find a counter-example for the case $occ_u \le occ_v$. The reasoning is analogous for the second case.

Let $a$, $b$ and $c$ be three contigs in $\mathcal {C}$ such that all their identifiers are different, $mult_a = 2$, $mult_b = mult(c) = 1$, and $(a_f, b_f) \in \mathcal {L}{}$, $(b_f, a_f) \in \mathcal {L}{}$ and $(a_f, c_f) \in \mathcal {L}{}$.

Let $fa_1 = (i, j)$, $fa_2 = (i', j')$ be the two direct fragments associated to $a$ and $fb = (k, l)$, $fc = (m, n)$ the two respectively associated to $b$ and $c$. The idea behind the proof is to know if the adjacent direct fragments $fa_1 \, fb \, fa_2 \, fc$ can participate into a solution path $Path$. In such a case, $Path$ is sub-defined by edges $i \rightarrow k \rightarrow i' \rightarrow m$ (and $j \rightarrow l \rightarrow j' \rightarrow n$). Remark that $k$ can permute with $l$, and $m$ with $n$.

According to $ADirF$ set definition, $(i, k) \in ADirF{}$, $(k, i') \in ADirF{}$ and $(i', m) \in ADirF{}$. If we constrain the occurrences by the relation $occ_u \le occ_v$, it implies that:

$$\begin{aligned}{}&occ_i \le occ_k (= occ_l - 1) \\&occ_l - 1 \le occ_{i'} - 1 \\&occ_{i'} \le occ_m \end{aligned}$$

As $$fa_1 \ne fa_2$$, $$occ_i \ne occ_{i'} - 1$$:

if $occ_i < occ_{i'} - 1$: then when we focus on the first edges path (in the “best” case $n$ permutes with $m$) $occ_k (= 0) \le occ_{i'} (= 2) \le occ_n (= 1)$, so it is absurd;
else $occ_i > occ_{i'} - 1$: then when we focus on the first edges path, you can permute $i'$ and $i$ and you fall into the same absurd implication as above.

$$\square$$

#### Proposition 16

(Occurences reduction for equal identifiers in $ADirF$) There exists an order between the occurrences of the vertices of an edge between two direct fragments that does not reduce the number of feasible and distinct solutions when the vertex’ identifiers are equal.

#### Proof

Let $u_1$, $u_2, \dots , u_n$, $n = 2 \left\lfloor \frac{mult_u}{2}\right\rfloor$ be same identifiers vertices participating in a solution path for $\mathcal {IRP}$, in this order (i.e. $\forall k \in \llbracket {1, n}\llbracket$, $u_k$ is before $u_{k+1}$ in the solution path).

It can be proven^12^ that: regardless the equation set of $k-1$ order relation $R_{k,k+1} \in \{<, >\}$ between two vertices $ctg_{uk}$ and $ctg_{uk+1}$ (i.e. $ctg_{uk} R_{k,k+1} ctg_{uk+1}, \forall k \in \llbracket {1, n}\llbracket$), there exists a permutation between all distinct occurrences of $u$ such that all the relations $R_{k,k+1}$ are satisfied. Thus, let defined the following equation set (used in $ADirF$):

$$\begin{aligned} R_{k,k+1}:= {\left\{ \begin{array}{ll} < &{} \text {if } or_{uk} = 0 \vee or_{uk+1} = 0 \\ > &{} \text {else } or_{uk} = or_{uk+1} = 1 \end{array}\right. } \end{aligned}$$

Have we got enough edges to build a path with $\frac{n}{2}$ adjacent direct fragments? On the one hand, the maximum number of edges we would need is: $2 \left( {\left\lfloor {\frac{mult_u}{2}}\right\rfloor - 1}\right)$ (the $2$ is caused by direct fragments). On the other hand, $ADirF$ provides:

$$\begin{aligned} 2 \sum _{k' = 1}^{\left\lfloor {\frac{mult_u}{2}}\right\rfloor - 1} 1 = 2 \left( {\left\lfloor {\frac{mult_u}{2}}\right\rfloor - 1}\right) \end{aligned}$$

edges, (the $2$ is caused by diradj function). $\square$

#### Proposition 17

(Canonical reductions for $ADirF$ for equal identifiers) $ADirF$ set contains only one of the eight edges that exist between two adjacent direct fragments when the vertex’ identifiers are equal, i.e.

$$\forall (i, j) \in DirF{}, \forall (k, l) \in DirF{} \mid ctg_i = ctg_k \wedge occ_i < occ_k$$:

$$\begin{aligned} \begin{array}{l} (i, k) \in E \\ \iff (i, l) \in E \\ \iff (j, k) \in E \\ \iff (j, l) \in E \end{array}&\iff \begin{array}{l} (i, k) \in ADirF{} \\ \wedge (\overline{k}, \overline{i}) \notin ADirF{} \\ \wedge (i, l) \notin ADirF{}\\ \wedge (\overline{l}, \overline{i}) \notin ADirF{} \\ \wedge (j, k) \notin ADirF{}\\ \wedge (\overline{k}, \overline{j}) \notin ADirF{} \\ \wedge (j, l) \notin ADirF{}\\ \wedge (\overline{l}, \overline{j}) \notin ADirF{} \end{array} \\\\ \begin{array}{l} (k, i) \in E \\ \iff (k, j) \in E \\ \iff (l, i) \in E \\ \iff (l, j) \in E \end{array}&\iff \begin{array}{l} (k, i) \notin ADirF{} \\ \wedge (\overline{i}, \overline{k}) \in ADirF{} \\ \wedge (k, j) \notin ADirF{}\\ \wedge (\overline{j}, \overline{k}) \notin ADirF{} \\ \wedge (l, i) \notin ADirF{}\\ \wedge (\overline{i}, \overline{l}) \notin ADirF{} \\ \wedge (l, j) \notin ADirF{}\\ \wedge (\overline{j}, \overline{l}) \notin ADirF{} \end{array} \end{aligned}$$

#### Proof

The reciprocals are trivial as $ADirF{} \subset E$.

Only $(i, k) \in ADirF{}$ and $(\overline{i}, \overline{k}) \in ADirF{}$ as the occurrences of $i$, $\overline{i}$, $k$ or $\overline{k}$ are even, and $occ_i = occ_{\overline{i}} < occ_k = occ_{\overline{k}}$.

For the other cases, either they contradict the even-occurrences constraint or the constraint on the occurrences order. $\square$

Reminder of adjacent inverted fragments set definition:

$$\begin{aligned} AInvF{}= & {} {}&\left\{ \begin{aligned}{}&(u, v) \in E \text { s.t.}\\&\quad ctg_u< ctg_v \\&\quad \wedge occ_u = 2 k + or_u\\&\quad 0 \le k< \left\lfloor {\frac{mult_u}{2}}\right\rfloor \\&\quad \wedge occ_v = 2 k' + or_v \\&\quad 0 \le k'< \left\lfloor {\frac{mult_v}{2}}\right\rfloor \\ \end{aligned} \right\} \\&\bigcup{} & {} \left\{ \begin{aligned}{}&(u, v) \in E \text { s.t.} \\&\quad ctg_u = ctg_v \\&\quad \wedge (or_u = f \vee or_v = f)\\&\quad \wedge occ_u - or_u = 2 k \\&\quad \wedge occ_v - or_v = 2 k'\\&\quad 0 \le k< k' < \left\lfloor \frac{mult_u}{2}\right\rfloor \end{aligned} \right\} \end{aligned}$$

This set is the minimum exhaustive set of canonical edges which represent adjacent inverted fragments. Two combinatorial reductions are necessary—*canonical reduction for different identifiers* (Propositions 18 and 19) and *same identifiers occurrences* reductions (Propositions 20 and 21).

#### Proposition 18

(Canonical reductions for $AInvF$ for different identifiers) $AInvF$ set contains only one of the four edges that exist between two adjacent inverted fragments when the vertex’ identifiers are different, i.e.

$$\forall (i, j) \in InvF{}, \forall (k, l) \in InvF{} \mid ctg_i < ctg_k$$:

$$\begin{aligned} (i, k) \in E&\iff \begin{array}{l} (i, k) \in AInvF{} \\ \wedge (\overline{k}, \overline{i}) \notin AInvF{} \\ \wedge (l, j) \notin AInvF{}\\ \wedge (\overline{j}, \overline{l}) \notin AInvF{} \end{array} \\\\ (k, i) \in E&\iff \begin{array}{l} (k, i) \notin AInvF{} \\ \wedge (\overline{i}, \overline{k}) \notin AInvF{} \\ \wedge (j, l) \in AInvF{} \\ \wedge (\overline{l}, \overline{j}) \in AInvF{} \end{array} \\\\ (i, l) \in E&\iff \begin{array}{l} (i, l) \in AInvF{} \\ \wedge (k, j) \notin AInvF{}\\ \wedge (\overline{l}, \overline{i}) \notin AInvF{} \\ \wedge (\overline{j}, \overline{k}) \notin AInvF{} \end{array} \\\\ (j, k) \in E&\iff \begin{array}{l} (j, k) \in AInvF{} \\ \wedge (\overline{k}, \overline{j}) \notin AInvF{} \\ \wedge (l, i) \notin AInvF{}\\ \wedge (\overline{i}, \overline{l}) \notin AInvF{} \end{array} \end{aligned}$$

#### Proof

The reciprocals are trivial as $AInvF{} \subset E$.

Let $(i, j) \in InvF{}$, $(k, l) \in InvF{}$ be two adjacent inverted fragments such that $ctg_i < ctg_k$. We will focus on demonstrating the case $(i, k) \in E$. The others follow the same logics.

- $(i, k) \in AInvF{}$ as $\exists n \in \llbracket {0,\left\lfloor \frac{mult_i}{2}\right\rfloor }\llbracket$ such that $occ_i = 2n + or_i = 2n$, and $\exists n' \in \llbracket {0,\left\lfloor \frac{mult_k}{2}\right\rfloor }\llbracket$ such that $occ_k = 2n' + or_k = 2n' + 1$;
- $$(\overline{k}, \overline{i}) \notin AInvF{}$$ as $$ctg_{\overline{k}} > ctg_{\overline{i}}$$;
- $$(l, j) \notin AInvF{}$$ as $$ctg_l > ctg_j$$;
- $(\overline{j}, \overline{l}) \notin AInvF{}$ as $or_{\overline{j}} = 0$ and $occ_{\overline{j}} \not \mid 2$ so $\not \exists n \in \llbracket {0,\left\lfloor \frac{mult_j}{2}\right\rfloor }\llbracket$ such that $occ_{\overline{j}} = 2n$.

$$\square$$

#### Proposition 19

(Minimum $AInvF$ set for different identifiers) When the vertex’ identifiers are different, it is not possible to reduce the number of canonical edges based on occurrences comparisons.

#### Proof

In order to reduce $AInvF$ when the vertex’ identifiers are different, we have to be more restrictive on the occurrences constraints. To reduce the loops on $k$ and $k'$, we have to find an order between the occurrences of $u$ and $v$.

However, for each order relation between the occurrences ($occ_u \le occ_v$ and $occ_u \ge occ_v$), we can find two associated counter-examples. For the sake of clarity, we will find a counter-example for the case $occ_u \le occ_v$. The reasoning is analogous for the second case.

Let $c$ and $d$ be two contigs in $\mathcal {C}{}$ such that $ctg_c < ctg_d$, $mult(c) = 4$ and $mult_d = 2$, and $(c_f, d_f) \in \mathcal {L}{}$ and $(d_f, c_f) \in \mathcal {L}{}$. Let $p_1 = (i, j)$, $p_2 = (i', j')$ be the two inverted fragments associated to $c$ and $q = (k, l)$ the one associated to $d$. The idea behind the proof is to know if the adjacent inverted fragments $p_1 \, q \, p_2$ can participate into a solution path $Path$. In such a case, $Path$ is sub-defined by edges $i \rightarrow k \rightarrow i'$ and $j' \rightarrow l \rightarrow j$.

According to $AInvF$ set definition, $(i, k) \in AInvF{}$ and $(j', l) \in AInvF{}$. If we constrain the occurrences by the relation $occ_u \le occ_v$, thus on the one hand, $occ_i \le occ_k (= occ_l - 1)$, and on the other hand $occ_l - 1 \ge occ_{j'} - 1$. As $p_1 \ne p_2$, $occ_i \ne occ_{j'} - 1$:

if $occ_i < occ_{j'} - 1$: then $occ_k (= 0) > occ_i (= 0)$ so it is absurd;
else $occ_i > occ_{j'} - 1$: then $occ_i (= 2) \le occ_k (= 0)$ so it is absurd.

$$\square$$

#### Proposition 20

(Occurences reduction for equal identifiers in $AInvF$) There exists an order between the occurrences of the vertices of an edge between two inverted fragments that does not reduce the number of feasible and distinct solutions when the vertex’ identifiers are equal.

#### Proof

Let $u_1$, $u_2, \dots , u_n$, $n = 2 \left\lfloor \frac{mult_u}{2}\right\rfloor$ be same identifiers vertices participating in a solution path for $\mathcal {IRP}$, in this order (i.e. $\forall k \in \llbracket {1,n}\llbracket$, $u_k$ is before $u_{k+1}$ in the solution path).

It can be proven^13^ that: regardless the equation set of $k-1$ order relation $R_{k,k+1} \in \{<, >\}$ between two vertices $ctg_{uk}$ and $ctg_{uk+1}$ (i.e. $ctg_{uk} R_{k,k+1} ctg_{uk+1}, \forall k \in \llbracket {1, n}\llbracket$), there exists a permutation between all distinct occurrences of $u$ such that all the relations $R_{k,k+1}$ are satisfied. Thus, let define the following equation set (used in $AInvF$):

$$\begin{aligned} R_{k,k+1}:= {\left\{ \begin{array}{ll} < &{} \text {if } or_{uk} = 0 \vee or_{uk+1} = 0 \\ > &{} \text {else } or_{uk} = or_{uk+1} = 1 \end{array}\right. } \end{aligned}$$

Have we got enough edges to build a path with $\frac{n}{2}$ adjacent inverted fragments? On the one hand, the maximum number of edges we would need is: $2 \left( {\left\lfloor {\frac{mult_u}{2}}\right\rfloor - 1}\right)$ (the $2$ is caused by inverted fragments). On the other hand, $AInvF$ provides:

$$\begin{aligned} 2 \sum _{k' = 1}^{\left\lfloor {\frac{mult_u}{2}}\right\rfloor - 1} 1 = 2 \left( {\left\lfloor {\frac{mult_u}{2}}\right\rfloor - 1}\right) \end{aligned}$$

edges, (the $2$ is caused by invadj function). $\square$

#### Proposition 21

(Canonical reductions for $AInvF$ for equal identifiers) $AInvF$ set contains only one of the four edges that exist between two adjacent inverted fragments when the vertex’ identifiers are different, i.e.

$$\forall (i, j) \in InvF{}, \forall (k, l) \in InvF{} \mid ctg_i = ctg_k \wedge occ_i < occ_k$$:

$$\begin{aligned} (i, k) \in E&\iff \begin{array}{l} (i, k) \in AInvF{} \\ \wedge (\overline{k}, \overline{i}) \notin AInvF{} \\ \wedge (l, j) \notin AInvF{}\\ \wedge (\overline{j}, \overline{l}) \notin AInvF{} \end{array} \\ \\ (i, l) \in E&\iff \begin{array}{l} (i, l) \in AInvF{} \\ \wedge (k, j) \notin AInvF{}\\ \wedge (\overline{l}, \overline{i}) \notin AInvF{} \\ \wedge (\overline{j}, \overline{k}) \notin AInvF{} \end{array} \\\\ (j, k) \in E&\iff \begin{array}{l} (j, k) \in AInvF{} \\ \wedge (\overline{k}, \overline{j}) \notin AInvF{} \\ \wedge (l, i) \notin AInvF{}\\ \wedge (\overline{i}, \overline{l}) \notin AInvF{} \end{array} \end{aligned}$$

${Nota\,bene}$: $(k, i) \in E$ case is not here because $(i, k)$ does not change the solution as their sequences are equal. This non-redundancy advantage is allowed by occurrence reduction in Proposition 20.

#### Proof

The reciprocals are trivial as $AInvF{} \subset E$.

Let $(i, j) \in InvF{}$, $(k, l) \in InvF{}$ be two adjacent inverted fragments such that $ctg_i = ctg_k$ and $occ_i < occ_k$. We will focus on demonstrating the case $(i, k) \in E$. The others follow the same logics.

- $(i, k) \in AInvF{}$ as $\exists n \in \llbracket {0,\left\lfloor \frac{mult_i}{2}\right\rfloor }\llbracket$ such that $occ_i = 2n + or_i = 2n$, and $\exists n' \in \llbracket {0,\left\lfloor \frac{mult_k}{2}\right\rfloor }\llbracket$ such that $occ_k = 2n' + or_k = 2n' + 1$;
- $$(\overline{k}, \overline{i}) \notin AInvF{}$$ as $$occ_{\overline{k}} > occ_{\overline{i}}$$;
- $$(l, j) \notin AInvF{}$$ as $$occ_l > occ_j$$;
- $(\overline{j}, \overline{l}) \notin AInvF{}$ as $or_{\overline{j}} = 0$ and $occ_{\overline{j}} \not \mid 2$ so $\not \exists n \in \llbracket {0,\left\lfloor \frac{mult_j}{2}\right\rfloor }\llbracket$ such that $occ_{\overline{j}} = 2n$.

$$\square$$

## Appendix C

**Metrics**

### C.1 Quast metrics

Quast metric descriptions can be found at https://quast.sourceforge.net/docs/manual.html#sec3.1. Here we adapt the description of the ones we use in this paper.

- # Misassemblies is the number of positions in the contigs (breakpoints) that satisfy one of the following criteria:
  - The left flanking sequence aligns over 1 kbp away from the right flanking sequence on the reference;
  - Flanking sequences overlap on more than 1 kbp;
  - Flanking sequences align to different strands.
- # Local misassemblies is the number of positions in the contigs (breakpoints) that satisfy the following conditions:
  - The gap or overlap between left and right flanking sequences is less than 1 kbp, and larger than 200 bp (the maximum indel length);
  - The left and right flanking sequences both are on the same strand.
- Genome fraction (%) is the percentage of aligned bases in the reference genome. A base in the reference genome is aligned if there is at least one contig with at least one alignment to this base. Contigs from repetitive regions may map to multiple places, and thus may be counted multiple times

## Appendix D

**Results**

See Tables 8, 9, 10

**Table 8:** Benchmark 3 v1 contig quast

Instance	ILPs	%gnm	#mis	
Abies_alba	dr-sc	99.318	0	0
	ir-sc	99.318	0	0
Acorus_americanus	ir-sc	99.518	0	0
Agathis_dammara	dr-ir-sc	80.194	0	0
	ir-dr-sc	80.194	0	0
Azima_tetracantha	ir-sc	99.93	0	0
Begonia_pulchrifolia	–	–	–	–
Carpodetus_serratus	ir-sc	98.605	0	0
Circaeaster_agrestis	ir-sc	99.564	0	0
Clematis_repens	ir-sc	99.929	0	0
Commiphora_foliacea	ir-sc	98.336	0	0
Cucumis_hystrix	ir-sc	99.492	0	0
Eucommia_ulmoides	ir-sc	99.01	0	0
Jasminum_tortuosum	ir-sc	99.767	0	0
Juniperus_scopulorum	sc	98.822	0	0
Lamprocapnos_spectabilis	dr-sc	35.105	0	0
Lathyrus_pubescens	dr-sc	98.203	0	0
	ir-sc	98.203	0	0
Lophocereus_schottii	sc	98.625	0	0
Musa_ornata	ir-sc	99.082	0	0
Oenothera_glazioviana	ir-sc	98.495	0	0
Pelargonium_nanum	ir-sc	96.039	0	0
Podocarpus_totara	sc	98.757	0	0
Porphyra_purpura	dr-sc	99.945	0	0
Sagittaria_trifolia	ir-sc	98.547	0	0
Sciadopitys_verticillata	dr-sc	96.236	0	0
	ir-sc	96.236	0	0
Sciaphila_densiflora	sc	99.874	0	0
Selaginella_kraussiana	dr-sc	99.838	0	0
Selaginella_vardei	dr-sc	99.984	0	0
Taxus_baccata	sc	98.072	0	0
Triosteum_pinnatifidum	ir-dr-sc	98.298	0	0
Uvaria_macrophylla	ir-sc	98.347	0	0
Welwitschia_mirabilis	ir-sc	99.167	0	0
Wolffia_australiana	ir-sc	99.827	0	0

**Table 9:** benchmark 3 v1 ilp stats

Instance	$\mathsf {|{}\mathcal {C}{}|}$	$\mathsf {|{}\mathcal {L}{}|}$	$\mathsf {|{}V|}$	$\mathsf {|{}E|}$	Time
Abies_alba	9	28	20	36	0.02
					0.02
Acorus_americanus	5	16	16	40	0.04
					0.05
Agathis_dammara	20	54	50	96	0.2
					0.64
Azima_tetracantha	7	20	16	28	0.0
					0.0
Begonia_pulchrifolia	6	14	12	14	0.0
					0.0
Carpodetus_serratus	13	44	36	76	0.14
					0.21
Circaeaster_agrestis	13	36	44	112	0.37
					4.06
Clematis_repens	6	16	18	38	0.04
					0.04
Commiphora_foliacea	16	38	38	58	0.04
					0.04
Cucumis_hystrix	9	22	22	40	0.02
					0.03
Eucommia_ulmoides	13	32	32	52	0.03
					0.05
Jasminum_tortuosum	10	26	30	68	0.07
					0.13
Juniperus_scopulorum	10	28	20	28	0.0
					0.0
Lamprocapnos_spectabilis	12	36	26	46	0.0
					0.0
Lathyrus_pubescens	17	44	36	52	0.02
					0.02
Lophocereus_schottii	12	36	24	36	0.0
					0.0
Musa_ornata	15	32	46	82	0.24
					0.87
Oenothera_glazioviana	11	30	30	56	0.07
					0.08
Pelargonium_nanum	11	32	34	108	0.17
					0.43
Podocarpus_totara	13	42	26	42	0.0
					0.03
Porphyra_purpura	3	8	8	16	0.0
					0.0
Sagittaria_trifolia	15	32	34	46	0.17
					0.02
Sciadopitys_verticillata	25	70	52	78	0.02
					0.04
Sciaphila_densiflora	4	8	8	8	0.0
					0.0
Selaginella_kraussiana	5	12	14	26	0.01
					0.01
Selaginella_vardei	3	8	8	16	0.0
					0.0
Taxus_baccata	17	42	34	42	0.0
					0.0
Triosteum_pinnatifidum	13	40	32	66	0.07
					0.15
Uvaria_macrophylla	13	30	42	86	0.24
					0.84
Welwitschia_mirabilis	13	34	30	30	0.02
					0.03
Wolffia_australiana	10	28	24	44	0.02
					0.02

**Table 10:** benchmark 3 v2 ilp stats

Instance	$\mathsf {|{}\mathcal {C}{}|}$	$\mathsf {|{}\mathcal {L}{}|}$	$\mathsf {|{}V|}$	$\mathsf {|{}E|}$	Time
Agathis_dammara	20	54	52	108	0.22
					0.83
Begonia_pulchrifolia	6	14	14	22	0.0
					0.0
Carpodetus_serratus	13	42	36	74	0.13
					0.2
Jasminum_tortuosum	10	28	30	70	0.08
					0.13
Lamprocapnos_spectabilis	12	36	48	180	0.68
					2.67
Lathyrus_pubescens	17	44	36	52	0.02
					0.02
Lophocereus_schottii	12	34	24	34	0.0
					0.0
Pelargonium_nanum	11	32	36	128	0.33
					0.84
Podocarpus_totara	13	40	26	40	0.0
					0.0
Triosteum_pinnatifidum	13	40	32	66	0.07
					0.18

## Footnotes

1 The notation $x[i]$ denotes the $i$th; element in $x$. Indices start from $0$.

2 https://khloraa-scaffolding.readthedocs.io/en/latest.

3 To reproduce the results, please refer to https://khloraascaf-results.readthedocs.io/en/latest/benchmark_4/.

4 To reproduce the results, please refer to https://khloraascaf-results.readthedocs.io/en/latest/benchmark_5/.

5 For more details, please refer to https://khloraascaf-results.readthedocs.io/en/latest/benchmark_3/

6 CpGDB: A Comprehensive Database of Chloroplast Genomes http://www.gndu.ac.in/CpGDB/index.aspx

7 NCBI: The National Center for Biotechnology Information advances science and health by providing access to biomedical and genomic information https://www.ncbi.nlm.nih.gov/

8 For more details on the result for the initial version of the synthetic data, please refer to https://khloraascaf-results.readthedocs.io/en/latest/benchmark_3/v1_order_analysis/ and https://khloraascaf-results.readthedocs.io/en/latest/benchmark_3/v1_quast_analysis/.

9 For more details, please refer to https://khloraascaf-results.readthedocs.io/en/latest/benchmark_3/synthetic_data_v2/.

10 For more details on the results for the cleared version of the synthetic data, please refer to https://khloraascaf-results.readthedocs.io/en/latest/benchmark_3/v2_order_analysis/ and https://khloraascaf-results.readthedocs.io/en/latest/benchmark_3/v2_quast_analysis/.

11 https://pypi.org/project/khloraascaf/.
